# Decision-making pathways for adopting, implementing, and scaling gamified physical activity interventions in municipal policy and practice in the Netherlands

**DOI:** 10.1371/journal.pdig.0001792

**Published:** 2026-10-26

**Authors:** Ayla Schwarz, Maarten van Bottenburg, Monique Simons

**Affiliations:** 1 Consumption & Healthy Lifestyles, Wageningen University, Wageningen, the Netherlands; 2 Utrecht School of Governance, Utrecht University, Utrecht, the Netherlands; Peking University First Hospital, CHINA

## Abstract

Municipalities increasingly pilot gamified physical activity (PA) interventions, yet few progress beyond pilots. This qualitative study examined decision-making pathways through which Dutch municipalities adopt, implement, and scale digital gamified PA interventions, using the case of ‘Missie Master’. A two-phase design was applied, combining exploratory interviews with policymakers, health professionals, and a company (phase 1), followed by two multi-stakeholder workshops and follow-up interviews with municipalities new to gamification (phase 2). Thematic analysis showed that adoption was driven by informal processes, enthusiastic individuals, trust in product reliability, and peer recommendations, while generic company outreach was ignored. Implementation relied on early preparatory capacity, such as designated coordinators, marketing plans, and timely app updates. Scaling was rare and cyclical, dependent on political support, cross-domain alignment, and short communication lines. The study offers a practical decision guide and highlights monitoring needs. Policy implications include transparent evaluation pathways, structured early implementation, and strengthened public-private co-development.

## Author summary

Municipalities increasingly experiment with digital tools that use game elements to encourage, especially children, to be more physically active. While many of these initiatives show promise, most remain small pilot projects and do not become part of long-term local policy or everyday practice. In this study, we wanted to understand why this happens and what helps municipalities move beyond experimentation. We studied how Dutch municipalities decided whether to adopt, use, and continue a gamified physical activity app. By talking with policymakers, health professionals, and an app developer, we explored how decisions were made in practice. We found that adoption often depended on enthusiastic individuals and informal recommendations rather than formal evaluation procedures. Successful implementation required early preparation, such as clear coordination, communication, and ongoing support. Scaling initiatives beyond pilots proved difficult and usually depended on political support, cooperation across policy domains, and close collaboration between those making policy and those delivering activities. Our findings show that moving from pilots to sustained policy requires clearer decision pathways, better preparation, and stronger collaboration between public and private partners. These insights can help municipalities make more effective use of digital health innovations in the future.

## Introduction

Gamification has emerged as a promising strategy to enhance engagement and participation in digital health interventions [1,2]. By incorporating game elements into physical activity (PA) interventions, gamification seeks to foster sustained behavioral change and promote PA by making participation more engaging and enjoyable [3–5]. These strategies align with the preferences of youth [6–8] and have demonstrated potential for promoting PA across diverse populations [9,10]. However, despite increasing evidence and availability of gamified PA interventions, their integration into sustained municipal policy and routine practice remains limited, with many initiatives limited to short-term pilots and restricted public health impact [11–13], whereas the real-world processes of adoption, implementation, and scaling within municipal contexts remain underexplored [14]. In the Netherlands, municipal stakeholders, including policymakers and health professionals (e.g., sport coaches), have explicitly requested clear, evidence-based guidelines for evaluating and integrating gamified PA interventions into local policy. This aligns closely with the global action plan by the WHO (i.e., action 4.3), which emphasizes the importance of digital technologies and innovation to accelerate the development, adoption, implementation, and scaling of effective PA policy solutions [15].

### Adoption challenges

Adoption refers to the behavioral intention to use an intervention [16], a process that requires clear decision-making pathways and supportive approval frameworks to guide municipalities in their choices [17]. While platforms like the Dutch ‘GGD App Store’ provided some adoption support for evaluating app quality, no national approval framework exists for non-medical health apps. As a result, adoption decisions are challenging and inconsistent [18]. This reflects a broader international tension, where the rapid growth of commercially available health apps has not been accompanied by clear and well-operationalized evaluation criteria or standardized decision pathways. As a result, policymakers lack standardized frameworks to support consistent and informed adoption decisions [17,18]. Technological advancements are rapidly outpacing scientific evaluation, with new apps often lacking rigorous testing [19]. An increasing number of gamified PA interventions are being developed [2], but few have been effectively tested first and scaled afterwards [20]. Testing and scaling remain time-consuming and complex processes [21], however time differs between approximately five years [22] to requiring 14–17 years from development to widespread adoption [22,23]. The absence of clear and transparent evaluation pathways, clarifying what evidence is required, who evaluates health apps, and how decisions are made, also makes it difficult for companies (e.g., digital technology developers) seeking to expand beyond initial target groups or local contexts to align product development with municipal policy priorities [17] or in navigating the adoption landscape across municipalities [24]. In addition, municipalities face practical challenges such as limited staff capacity, technical problems during use, and competing policy priorities that further complicate implementation in daily practice [14].

### Implementation challenges

Although some Dutch municipalities and executing sports organizations, and respectively policymakers and health professionals (i.e., intervention implementers) have supported gamified digital PA interventions, these efforts are typically fragmented, small-scale, and lack strategies for sustainable implementation. As a result, promising initiatives frequently fail to progress beyond pilot stages and do not achieve structural policy integration. We define implementation here as the actual use and integration of an intervention into daily practice [16]. Health professionals such as neighborhood sport coaches (NSCs; in Dutch *buurtsportcoach*) who are, among others, responsible for executing the municipal sport policies, frequently report insufficient financial and organizational support from policymakers, further limiting the long-term impact of these initiatives and hampering adoption and implementation [14]. Many promising interventions never move beyond pilot stages [24–26], creating a cycle of small-scale pilot projects, remaining stuck in the early adoption stage and with limited opportunities for scaling and sustainable policy integration [27]. Hence, impact on public health is being hindered.

### Scaling challenges

Once interventions are adopted and implemented, the next challenge is how to sustain and expand them through scaling to optimize impact on public health. Scaling existing, effective gamified health interventions represents a more cost-effective and sustainable alternative to repeatedly developing new ones [27]. Investing in the scaling of proven interventions enables refinement, ongoing improvement, and the integration of adaptive elements to meet diverse user needs while maintaining efficiency [28]. This approach concentrates resources on optimizing a single intervention to maximize impact across different contexts, rather than fragmenting efforts across multiple short-term interventions [27]. Despite the importance of scaling, research on scaling gamified health interventions remains scarce. This scarcity limits guidance for policymakers on how to move from experimentation to systemic integration. When deciding whether to scale interventions, policymakers typically consider several criteria. Effectiveness is considered essential and usually serves as the primary prerequisite for further consideration. Cost-effectiveness is also valued, although it may be weighed differently depending on the context. Beyond these, decision-makers increasingly consider additional factors such as user acceptability, equity, and alignment with local priorities [17,29]. Effective scaling is further complicated by the need to adapt interventions to diverse local contexts where needs and resources can differ substantially [21,29]. However, the relative importance assigned to these factors and the value systems guiding these trade-offs are often unclear or inconsistent across decision-making contexts, and decisions are frequently shaped by immediate policy priorities, available resources, and individual experience rather than solely by intervention effectiveness [17]. As [17] highlight, the lack of transparent, consistent value frameworks makes it difficult to make informed, aligned decisions for policymakers and health professionals on the one hand and companies on the other. This gap is also evident in the gamification field, where research on how value-based decisions are made within municipal policy settings is scarce.

Emerging literature in the public sector suggests that scaling innovations is a complex, multifaceted process [30]. Although several conceptual frameworks and step-by-step guides for scaling health innovations exist, these often lack practical assessment tools that policymakers and practitioners can directly apply in routine decision-making [31].

Within public health research, scaling is predominantly conceptualized as ‘*scaling up’*, referring to the deliberate expansion of proven interventions to reach larger populations or broader systems. As a result, other dimensions of scaling receive less explicit attention. In contrast, the public sector innovation literature categorizes scaling into five distinct dimensions, each addressing different aspects of broadening the impact of innovations: scaling out, scaling up, scaling deep, and scaling beyond.

Scaling *out* refers to the geographical expansion or replication of an initiative, allowing its implementation across different locations [30], which has been earlier defined as horizontal scale-up [26]. Scaling *in* focuses on deepening the integration of an innovation within a specific area or organization, ensuring its sustainability and effectiveness [32]. Scaling *up* involves embedding innovations within policy frameworks or agendas to ensure long-term systemic change [30], earlier defined as vertical scale-up [26]. Scaling *deep* aims to institutionalize cultural values, fostering long-term shifts in systems and communities [32]. Finally, scaling *beyond* seeks to transfer innovations into entirely new fields or sectors [32].

In this study, we focus on three primary scaling approaches: 1) scaling *in*, integrating a gamified PA intervention within sport organizations where health professionals work, responsible for executing the municipal sport agreement and implementing the intervention (i.e., perspective of health professionals), 2) scaling *out*, replicating a gamified PA intervention developed for one municipality in the Netherlands across multiple municipalities in the Netherlands (i.e., perspective of companies), and 3) scaling *up*, embedding a gamified PA intervention within municipal policies to achieve systemic change (i.e., perspective of policymakers).

### Aim

Although gamified PA apps show promise, municipalities lack structured evaluation pathways and early preparatory supports (e.g., coordinators) to translate pilots into sustained policy and practice. Little is known about how municipalities adopt, implement, and scale gamified PA interventions within real-world policy contexts. While effectiveness and cost-effectiveness are often considered in decision-making, the value systems guiding other factors, such as acceptability, equity, and sustainability, remain unclear [17]. As [17] argues, when health systems clarify how innovations will be assessed and valued, companies can better align product development to meet policy needs, thereby closing the policy-company evidence gap. Furthermore, research rarely addresses how policymakers, health professionals, and companies can collaboratively scale effective interventions across diverse contexts. Therefore, this study aims to explore the decision-making pathways from adoption, to implementation, and scaling mechanisms that shape whether gamified PA apps transition from pilots to sustained municipal policy and practice.

This study uses a case study focusing on the digital gamified PA intervention ‘Missie Master’. ‘Missie Master’ was designed by 8D, a Dutch company specialized in design-oriented and co-creative projects. The app was developed to promote physical activity among children aged 8–12 years (S1 Text) within their local neighborhood. It encourages children to complete neighborhood-based missions involving physical activities and rewards them through the app for their participation. The app enables primarily health professionals or implementers, such as NSCs, as well as other coaches or teachers to create these interactive PA missions (e.g., neighborhood scavenger hunts and quizzes). Rather than evaluating the app itself, the study examines the adoption, implementation, and scaling process to generate practice-oriented insights of how municipalities move from pilot experimentation to policy and practice integration of gamified PA interventions.

### Theoretical underpinning

Two complementary frameworks are integrated to analyze how municipalities adopt, implement, and scale gamified PA interventions from early interest to routine policy and practice: The Exploration, Adoption/Preparation, Implementation, and Sustainment (EPIS) framework and the Validated Scaling Instrument.

The EPIS framework was selected because it is a widely used implementation science framework that captures implementation as a phased process and considers the multilevel contextual factors influencing adoption, implementation, and sustainment [33]. However, recent scholarship has highlighted the need for greater attention to policy processes and institutionalization within implementation science frameworks, including EPIS [34]. As municipalities play a central role in translating local health initiatives into sustainable policy and practice, implementation perspectives alone were insufficient for the purposes of this study.

Therefore, we complemented EPIS with the Validated Scaling Instrument, which was specifically developed to understand the scaling of public-sector innovations and distinguishes between emergence, institutionalization, and stabilization as scaling phases. The instrument also provides explicit attention to scaling strategies, including scaling in, scaling out, and scaling up, which are particularly relevant for municipalities seeking to move beyond pilot implementation toward broader institutional and policy integration [32]. Together, these frameworks offer a comprehensive lens for understanding both implementation processes and scaling dynamics within municipal contexts. They offer municipalities, including policymakers and health professionals, a clear phased outline to translate local experimentation into sustainable policy integration. This is particularly important because the municipalities’ decisions determine whether gamified PA interventions remain pilots or become embedded in routine policy. Table 1 maps our study focus onto phases in both frameworks.

**Table 1:** Conceptual framework to assess adoption, implementation, and scaling.

Study focus	EPIS phase(s)	Scaling phase(s)	Study application
Adoption	Exploration; Adoption/Preparation	Emergence	Intention and decision to use, including early piloting and initial organizational alignment.
Implementation	Implementation	Institutionalization	Integration into routines, roles, and budgets within municipal and partner organizations.
Scaling	Sustainment	Stabilization	Long-term routinization and policy embedding.

#### Adoption: exploration, adoption/preparation & emergence phase.

Within EPIS, *Exploration* refers to organizations becoming aware of a problem or improved approach and scanning for options and fit. *Adoption/Preparation* covers the decision to adopt and the preparatory work for active implementation. Adoption decisions are influenced by multi-level outer (e.g., policy, funding, interorganizational networks) and inner (e.g., leadership, climate, resources) contextual factors and may include pilot experimentation before a formal decision [33]. In the Validated Scaling Instrument, this stage corresponds to *emergence*. The innovation begins to break out of its niche, with diffusion and self-organization enabling the creation of new structures, routines, and organizational forms that can act as “niche-regimes” [32]. In this study, *adoption* refers to the behavioral intention and commitment to use the intervention while stakeholders acquire and process information. This includes individual uptake (e.g., downloading an app) [35], organizational commitment (e.g., integrating the tool into local organizational structures), and municipal commitment (e.g., providing pilot funding), which highlights the policy pathways through which early interest is translated into formal municipal action.

#### Implementation: implementation & institutionalization phase.

In EPIS, *Implementation* involves aligning outer-context enablers (e.g., funding, contracting, interorganizational networks) and inner-context factors (e.g., structure, culture/climate, leadership, innovation–values fit, workforce readiness) to support effective delivery, monitoring, and feedback [33]. Within the Validated Scaling Instrument, this stage corresponds to *institutionalization*, where implementation occurs at regime level, as actors build new alliances, norms, cultures, and routines around the innovation [32]. In this study, *implementation* refers to embedding the intervention into routine practice by relevant health professionals (i.e., implementers) [16].

#### Scaling: sustainment & stabilization phase.

In EPIS, *Sustainment* refers to the continued use of an intervention within stable organizational and system contexts, with ongoing monitoring, adaptations, and optimization. Sustainment reflects long-term commitment, supported by durable policies, budgets, and practices [33]. Within the Validated Scaling Instrument, this stage aligns with *stabilization*, where the innovation and its accompanying regime are tweaked and optimized to become the new status quo, often requiring additional institutional adjustments (e.g., policy or legal changes) and broad stakeholder support [32]. In this study, *scaling* is understood as the process by which municipalities move from localized implementation toward long-term stabilization of gamified PA interventions within their policies and structures. This includes durable integration into municipal plans and budgets, sustained engagement of professionals, and expansion across neighborhoods and schools. It is further in scaling in, referring to the integration of the gamified PA intervention within sport organizations where health professionals work and implement municipal sport agreements; scaling out, referring to the replication of the intervention across municipalities, as pursued by companies aiming for wider geographic reach; and scaling up, referring to embedding the intervention within municipal policy frameworks to stimulate systemic and long-term change.

Building on these complementary frameworks, we designed a two-phase study to explore how municipalities adopt, implement, and scale (in, out, and up) gamified PA interventions, and how their decisions shape the transition from short-term pilots to sustainable health policy and practice.

## Materials and methods

### Ethics statement

This study received ethical approval from the Social Sciences Ethics Committee of Wageningen University & Research (2023-087).

### Study design and study population

This study consisted of two phases: 1) exploratory interviews and 2) workshops. Phase 1 involved exploratory interviews with professionals from municipalities in the Netherlands that had prior experience in adopting, implementing, and scaling gamification, as well as with the CEO of a company in the Netherlands experienced in developing and scaling gamified interventions. Municipalities were identified using snowball sampling, supported by JOGG and Knowledge Centre for Sport & Physical Activity (KcSB, Dutch: Kenniscentrum Sport & Bewegen). JOGG is a Dutch public health organization that works towards a healthy lifestyle transition for youth. KcSB is a Dutch knowledge Centre that makes physical activity knowledge from science, policy, and practice available and applicable for professionals. Ultimately, four professionals representing three municipalities (municipalities A-C) participated. Insights from these interviews informed the setup of phase 2.

Phase 2 focused on the adoption, implementation, and scaling of the gamification PA intervention ‘Missie Master’ and consisted of two workshop sessions and one informal network session. Municipalities in the Netherlands without gamification experience were invited to participate in the workshops. In spring, JOGG published an invitation to participate in their newsletter (reaching 211 municipalities), municipalities were contacted via KcSB (reaching five municipalities), the company (reaching two municipalities), and the researcher’s professional network (e.g., colleagues and municipal partners from previous projects and collaborations, reaching 14 municipalities). In total, eight municipalities reacted, three declined due to a high workload before summer or their preference to develop an app from scratch. Eventually, five municipalities were willing to participate. Each municipality included at least one policymaker and one health professional (e.g., NSC). Although five municipalities began phase 2, only three completed the project (municipalities D-F). The first workshop was attended by five municipalities, in total consisting of 15 participants. The second workshop was attended by two municipalities, in total consisting of eight participants, with each municipality contributing two to four participants. Additionally, two participants from one municipality, who were unable to attend the second workshop, were interviewed separately.

### Procedure

#### Phase 1.

The exploratory interviews in phase 1 were conducted online between May and July 2023 via Microsoft Teams, each lasting approximately one hour. The exploratory interviews involved policymakers and health professionals (n = 4) from three municipalities with gamification experience, focusing on policy-making, the role of gamification in policy, support structures, and strategies for scaling gamification. Additionally, an interview was conducted with the participating company to understand their experience in scaling the ‘Missie Master’ app. This interview covered app development, scaling strategies, barriers and facilitators in scaling out, municipal responses with regard to ‘Missie Master’ and their experiences with scaling out other gamified interventions.

#### Phase 2.

Phase 2 consisted of the first workshop held in-person (April 2024), followed by an online network exchange meeting via Microsoft Teams (June 2024), and the second in-person workshop (September 2024). Since one municipality was unable to attend the second workshop, two additional interviews were conducted online (September 2024) via Microsoft Teams. All interviews and workshops were facilitated by the lead researcher and first author (AS) with support from a co-researcher and third author (MS) and research assistants (see acknowledgements). The timeline of the project is presented in Table 2.

**Table 2:** Overview of project phases, timeline, activities, and included stakeholders.

Phases	Phase 1	Phase 2				
Timeframe	May-July 2023	April 2024	April-September 2024	June 2024	September 2024	September 2024
Activity	Exploratory interviews	First workshop	Implementation of ‘Missie Master’ for 4 months	Informal network session	Second workshop	Interviews
Stakeholders	3 municipalities (including 4 policymakers/health professionals) 1 company (including one CEO of that offers the digital app)	5 municipalities (including at least one policymaker and one health professional per municipality) 1 company (including one CEO of that offers the digital app)	3/5 municipalities	2 municipalities (including at least one policymaker and one health professional per municipality) 1 company (including one CEO of that offers the digital app)	2 municipalities (including at least one policymaker and one health professional) 1 company (including one CEO of that offers the digital app) 2 knowledge institutes	One municipality (including one policymaker and one health professional) who were not present during the second workshop

The first workshop aimed to assess the innovation capacity of municipalities, draft an implementation plan for ‘Missie Master,’ and explore factors influencing the scaling of gamification. Participants completed a pre-workshop questionnaire on innovation capacity. During the workshop, policymakers and health professionals discussed their questionnaires’ results, followed by a presentation from the company outlining best implementation practices for ‘Missie Master’ based on their implementation experiences in other municipalities. Participants then drafted an implementation plan, and mapped potential barriers and strategies, including ‘inspiration cards’ (including barriers, facilitators, and strategies) based on preliminary findings from phase 1 for scaling gamification (S2 Text).

After following the workshop, the company provided municipalities access to ‘Missie Master’ (one account per municipality), allowing them to determine their own implementation strategies between April and September 2024.

An informal network meeting was held in June 2024 via Microsoft Teams, where municipalities shared their implementation progress and discussed challenges in scaling gamification. The company was present to address technical issues and other concerns.

The second workshop, held in September 2024, revisited municipalities’ public innovation capacity, reviewed ‘Missie Master’ implementation experiences, and discussed strategies for embedding gamified interventions into municipal policy. Representatives from JOGG and KcSB joined alongside the company, providing additional perspectives on media and gamification. In preparation, municipalities completed a follow-up questionnaire on innovation capacity and an implementation evaluation form.

### Instruments

Several instruments were used across the project phases to assess the adoption (i.e., exploration, adoption/preparation and emergence phase), implementation (i.e., implementation and institutionalization phase), and scaling (i.e., sustainment and stabilization phase) of gamified PA interventions (see Table 3). All materials are available in S3 Text.

**Table 3:** Instruments.

	Phase 1	Phase 2				
Instrument	Exploratory interviews	First workshop	Implementation of ‘Missie Master’ for 4 months	Informal network session	Second workshop	Interviews
The Framework for Scaling Social and Sustainable Initiatives [30]	✓					
Public Innovation Capacity Questionnaire [36]	✓	✓			✓	✓
Validated Scaling Instrument [32]	✓	✓			✓	✓
CFIR – Implementation plan [37]		✓				
CFIR – Implementation evaluation [37]					✓	✓

During the exploratory interviews (Phase 1), the Framework for Scaling Social and Sustainable Initiatives (van Lunenburg et al., 2020) provided a structure for assessing the roles of municipalities (e.g., institutional factors) and companies (e.g., actor characteristics, strategic choices, and impact). In addition, the Validated Scaling Instrument [32], the Public Innovative Capacity [36], and prior research on digital gamification implementation among health professionals [14] were used. Together, these frameworks emphasize municipalities’ capacity to move beyond project-based implementation toward institutional change. They provide insights for policy scaling decisions and distinguish between scaling in, scaling out, and scaling up [32].

In both the first and second workshops (Phase 2), municipalities completed the Public Innovation Capacity questionnaire (Meijer, 2019) as a pre-workshop instrument. This self-assessment tool allowed policymakers and health professionals to reflect separately on their municipality’s innovation readiness. The questionnaire measures five key functions, namely mobilizing, improvising, vitalizing, balancing, and coordinating providing insights into the municipality’s capacity to integrate gamified PA interventions.

Also, in the first and second workshops (Phase 2), we applied the Validated Scaling Instrument [32]. This instrument identifies scaling phases (emergence, institutionalization, stabilization), barriers to scaling, and potential strategic pathways. It guided workshop discussions by structuring the complexities of scaling through five critical questions. Policymakers and health professionals used the tool together to identify their municipality’s current position in the scaling process, reflect on barriers, and outline scaling strategies.

Following the workshops, municipalities developed a Consolidated Framework for Implementation Research (CFIR)-based implementation plan and a CFIR-based implementation evaluation [37], across five domains (i.e., Intervention Characteristics, Outer Setting, Inner Setting, Characteristics of Individuals, and Process) [37]. These instruments focused on key CFIR constructs, such as goals/feedback, relative advantage, leadership engagement, internal implementation leaders, external change agents, opinion leaders, compatibility, key stakeholders, available resources, and reflecting/evaluating. Together, these activities helped municipalities identify facilitators and barriers to implementing the intervention, while also helping municipal decision-makers anticipate the organizational adjustments needed for policy integration.

### Data analysis

In phase 1, all exploratory interviews were audio-recorded and transcribed verbatim by an external transcription service. The lead researcher (AS) reviewed all transcripts for accuracy and pseudonymization. The data was independently coded (BB) (with 10% double-coded by AS) and thematically analyzed (BB) using ATLAS.ti (version 23; ATLAS.ti Scientific Software Development GmbH) [38]. Identified facilitators, barriers, and strategies were used for the creation of ‘inspiration cards’ in phase 2. In phase 2, data of the first workshop included participants’ documentation on municipality-specific outcomes that was transferred from paper to digital format, while researchers’ notes, based on standardized observation plans (S4 Text), summarized the plenary discussions and key findings. The informal network meeting was documented by AS, while the second workshop was audio-recorded and transcribed verbatim by an external transcription service. The lead researcher (AS) reviewed all transcripts for accuracy and pseudonymization. Transcripts were coded (LD) (with 10% double-coded by AS) and thematically analyzed using ATLAS.ti (version 23; ATLAS.ti Scientific Software Development GmbH) [38]. A combination of inductive and deductive coding (S5 Text) approach was applied [39]. Deductive codes were based on the Scaling Social and Sustainable Initiatives [30], Public Innovation Capacity [36], and Validated Scaling Instrument [32]. In the end, both analyses of phase 1 and phase 2 were merged to overall themes, aligning with adoption, implementation, and scaling (Table 1).

## Results

Municipalities A-C had gamification experience (phase 1), and municipalities D-F had no gamification experience and implemented ‘Missie Master’ for the first time (phase 2). Any differences between municipalities with or without gamification experience are explicitly noted in the text. Municipalities were at varying stages of the scaling process. While municipalities without gamification experience (D–F) began in the adoption phase, they progressed toward the implementation phase by the end of the project, with their innovation capacity evolving. Municipalities with gamification experience (A-C) were in the implementing or scaling phase, but also reflected on the past adoption phase and future scaling phase. The following section outlines the three phases, adoption, implementation, and scaling.

### Adoption: exploration, adoption/preparation & emergence phase

Six sub-themes related to the adoption phase (Table 4).

**Table 4:** Identified facilitators and challenges in the adoption phase.

Sub-theme	Facilitators	Challenges
**Collaboration and stakeholder engagement**	• Engagement of key stakeholders • Clear communication of benefits and alignment with broader societal goals • Clear vision and enthusiasm to overcome skepticism • Knowledge exchange between municipalities • Trust in product reliability • Flexible app design allowed local tailoring • Joint development across municipalities	• Limited collaboration with private partners due to administrative burdens, lack of trust • Burden on municipalities to tailor app content • Preference for ownership and ‘reinventing the wheel’ • Unclear responsibility for adoption (policymakers vs. health professionals) (see Decision-making) • Variation in municipal policies and expectations
**Support and leadership**	• Enthusiastic individuals • Positive leadership attitudes among policy and executing PA organizations	• Negative leadership attitudes among policy and executing PA organizations • Resistance from schools, parents, and sports staff due to technical issues, limited device access, or restrictions
**Introductory meetings and pilot testing**	• Introductory sessions • Pilots enabled experimentation and showcased policy relevance	• Lack of starter packs, including an implementation and communication plan
**Alignment with policy goals**	• Gamification framed as a tool to achieve policy outcomes	• Rarely embedded as a core or long-term policy focus
**Sustaining adoption efforts**	• Gamification to reach inactive children, test in new neighborhoods, or explore inclusion in policy plans	• Timeline mismatch with municipal marketing • Poor fit for target group • Restricted school phone policies • Narrow reach • High preparation/promotion demands
**Decision-making**	• Personalized requests from the company tailored to municipal needs, local challenges, and policy priorities • Targeting enthusiastic and engaged individuals within municipalities • Recommendations from trusted colleagues • Consultation with other municipalities to share experiences and assess credibility	• Generic company outreach (e.g., standardized emails or promotional messages not tailored to the municipality) • Requests overlooked if not reaching engaged individuals • Uncertainty about product reliability • Need to verify alignment with local priorities and target groups

#### Collaboration and stakeholder engagement.

Health professionals and policymakers emphasized that engaging key stakeholders, such as local politicians, service providers, and other relevant partners, was important for gaining support. Clear communication of gamification’s benefits and alignment with broader societal goals helped to overcome skepticism, while knowledge sharing between municipalities strengthened adoption. For example, one municipality described its role as a testing ground, where both mistakes and successes provided lessons for other municipalities.

At the same time, collaboration was not always straightforward. Municipalities reported limited contact with private partners or hesitancy to engage due to administrative burdens and lack of trust, which slowed adoption. Trust in the company and confidence in product reliability (e.g., proven technology, sustained availability) were seen as prerequisites for integration. Flexibility in the app’s design enabled local tailoring but also increased the burden on municipalities to adapt content themselves. This led some municipalities to prefer developing their own tools, rather than adopting existing ones. Ownership expectations further varied. While some municipalities sought to retain control, others were open to joint development with other municipalities. Decision-making authority also differed, as in some cases policymakers were the primary decision-makers, whereas in others health professionals led adoption choices. As one company representative noted, “*every municipality had a different policy and was looking for something completely different in such an app*”, making it difficult to navigate multiple municipal policies.

#### Support and leadership.

Alongside structural collaboration, individual leadership and individual enthusiastic played an important role in adoption. Enthusiastic policymakers, aldermen, and health professionals were often key to initiating projects and sustaining momentum. Positive leadership attitudes within policy and executing PA organizations created room for experimentation and pilot testing, whereas negative or skeptical stances could halt innovation, even when other stakeholders were supportive. As one health professional noted: *“[…] if we’re enthusiastic we should just go try this and not be very reluctant […]. Sometimes you just have to be confident in doing something new as well.”* (Municipality C). Political leadership was also important, as an alderman’s personal interest in gamification can create strong backing: *At the time, it really helped that we had an alderman who was very interested in that. In any case, the alderman already saw gaming was upcoming.”* (Municipality A). Enthusiastic individuals within municipalities helped to legitimize gamification and frame it as a promising tool for policy goals. *“You really need to have someone who has to initiate that on their own initiative and who also must have a lot of patience for that, and not everyone is in that luxurious position of being able to do that, both financially and time-wise” (*Municipality A). At the same time, resistance from other stakeholders, such as schools, parents, or sports staff, often hindered adoption, particularly when traditional methods were preferred. Practical barriers such as login issues, limited access to mobile devices, or school restrictions on phone use added to this resistance.

#### Introductory meetings and pilot testing.

Municipalities without prior gamification experience emphasized the value of introductory meetings, where examples and app functionalities were explained. These sessions lowered entry barriers and created familiarity with the tool. However, the absence of a clear implementation and communication plan caused delays and required significant time investment from implementers, who needed to promote the app and engage participants. As one participant noted, *“The starter pack is missing (with a manual to get started properly) and a complete communication pack. How does the app work, what games are there… how does it work?”* (Municipality D). To address these uncertainties, municipalities adopted a hands-on approach by piloting the app on a small scale. These pilots provided valuable experiences that helped policymakers to see gamification’s potential to achieve policy objectives: *“These small pilots that we are doing now should ensure that the policymakers get more and more of an idea of ‘okay, this is also a way, for example, to get more people to move’.”* (Municipality F).

#### Alignment with policy goals.

Across municipalities, adoption was consistently framed in terms of achieving policy goals rather than as a policy focus in itself. Gamification was generally regarded as a tool to engage new or hard-to-reach groups and to promote PA in ways that traditional methods had failed to do. This framing made it easier to gain support from skeptical stakeholders: *“Gamification is just a really good tool to get a certain target group moving. Which previously didn’t work. So that’s how we see it and I’ve also noticed if I go from that angle, if I bring in a plan, that’s more likely to succeed.”* (Municipality F). However, while this framing facilitated adoption, it also meant that gamification was rarely embedded as a core or long-term policy priority.

#### Sustaining adoption efforts.

Not all municipalities were able to continue the project. Of the five municipalities that initially engaged in the project and completed the first workshop, only three completed the project. Two municipalities withdrew after the first workshop. One explained that project timelines did not align with their own marketing cycles, which required six to eight weeks of preparation, while another found the app too juvenile for their target group of secondary school students. The three municipalities that did continue saw gamification as a way to address specific needs, such as reaching inactive children, testing gamification in new neighborhoods, or exploring its inclusion in policy plans. At the same time, they faced obstacles, including targeting only a narrow group of children, new policies restricting phone use in schools, and the time required for preparation and promotion.

#### Decision-making.

Once companies scale out their products they approach municipalities, either policymakers or health professionals. When municipalities evaluate a new gamification proposed by a company, several questions and steps are considered by either the health professionals or policymakers. The steps outlined in Table 5 highlight the questions and considerations raised by both health professionals and policymakers when evaluating whether to adopt an innovation or not. A key factor identified is the role of the product owner in presenting the product. Requests from companies often arrive as generic outreach, such as standardized emails or promotional messages sent to multiple municipalities without reference to local priorities. These communications were perceived as less credible than personalized requests that explicitly addressed a municipality’s specific needs, policy goals, or local challenges, or recommendations from trusted colleagues within the municipality. The recipient of these requests is also critical. If the request does not reach an enthusiastic and engaged person, it is likely to be overlooked. Therefore, targeting the enthusiastic individuals is crucial to the success of these initiatives. Another important step involves municipalities frequently consulting with other municipalities. This serves two purposes. First, to learn from their experiences with the product and its implementation and second, to assess the credibility and reliability of the product or the company offering it. These considerations help ensure that the product aligns with local policies, is effective, and fits the needs of the target population.

**Table 5:** Decision guide for evaluating gamified intervention requests at municipality-level, by health professionals or policymakers.

Health professionals	Policymakers
**What is the goal of the gamification innovation?**	**Who is the owner of** **the gamification innovation?** Extern (e.g., company), versus intern (e.g., colleague)
	**How is the first contact (e.g., via mail) presented?** General versus specific mail tailored to the needs/goals of the municipality. Mail must include the purpose, match the municipality’s goals, an estimated cost/hours of commitment, and information about data management.
**Discussion among internal colleagues** Making plans together with community partners about implementing the intervention.	**Discussion among internal colleagues** - Policy review - Personal vision - Professional vision - Financial test
**Do we have the resources? Is it realistic?** Conversation within the municipality.	
**Contact company** Is the intervention ready/usable right away? - Yes: Does the intervention fit with NSCs and the policy? Is it easy to use? Is there a pilot/video of how it works? - No: Work with health professionals and the target group to develop the intervention. The intervention should match children’s experiences and be easy to use. Test it with children beforehand.	**Contact company**In discussion with the product owner about previous experiences and the effectiveness
**Contact other municipalities** Communicate with other municipalities (learn from each other).	**Contact other municipalities** In discussion with other municipalities and health professionals to check how it works in other municipalities as a reference to justify plans.
**How does the neighborhood feel about the product?** - Approach the intervention per district. - Is there any interest in the intervention (from children)? - Evaluate with children.	
**Implementation** Communicate with gym teachers about how the intervention will be set up. - Marketing: What are the communication tools? - Is there a toolkit on the intervention for starting the product?	
	**Offer from product owner**
	***Optional: inform aldermen dependent on project scale***

### Implementation: implementation and institutionalization phase

Five sub-themes were identified during the implementation phase among experienced (municipality A-C) and inexperienced (municipality D-F) municipalities (Table 6).

**Table 6:** Identified facilitators and challenges during the implementation phase.

Sub-theme	Facilitators	Challenges
**Time investment**	*•* Adapted to specific age groups or switched to comparable, user-friendly alternatives	*•* High time and energy demands *•* Technical problems and usability issues for children *•* Staff lacked skills to update content
**Marketing**	*•* Continuous/multiple marketing efforts *•* Smaller, neighborhood-focused approaches *•* Availability of communication and marketing materials	*•* City-wide campaigns
**Monitoring and proven outcomes**	*•* Combination of quantitative (downloads, missions played) and qualitative (children’s/teachers’ experiences) data	*•* Clear proven outcomes before committing to long-term support *•* Lack of long-term evidence
**Support and leadership**	*•* Political backing from aldermen *•* Enthusiastic individual	*•* Absence of enthusiastic individuals or driving forces *•* Support often guaranteed only for a limited period
**Policy landscape**	*•* Actively connecting gamification to emerging policy priorities	*•* Shifts in political leadership or policy focus

Implementation approaches varied (see Table 7), and while implementation created valuable opportunities, municipalities also reported significant challenges.

**Table 7:** Overview of the implementation of the movement app across the three municipalities.

	Municipality 1	Municipality 2	Municipality 3
**Target group**	*•* Children from 6 to 8 years old in low SEP neighborhoods	*•* Children from 8 to 12 years old	*•* Children from 8 to 12 years old
**Volume**	*•* Neighborhood-focused	*•* Citywide	*•* Neighborhood-focused
**Monitoring**	*•* Quantitative data: Number of participants, and when they visited the playground *•* Qualitative data: Experience children	*•* Quantitative data: Numbers and time of downloading; Missions played *•* Qualitative data: Enthusiastic colleagues	*•* Quantitative data: Number of times and time the app was played *•* Qualitative data: Experience children
**Execution**	*•* Schools	*•* Summer program	*•* Leisure time
**Led the intervention**	*•* Health professionals (i.e., NSC)	*•* Health professionals (i.e., NSC)	*•* Health professionals (i.e., NSC)
**External organizations involved**	*•* Schools *•* Youth and child workers	*•* None	*•* Schools *•* Communication partners
**Marketing**	*•* Flyers, posters in schools	*•* The summer program, verbal, and posters at a market stall	*•* Paid social media campaigns, promotion in schools, visibility in neighborhoods
**Support for neighborhood sports coaches**	*•* Time and budget	*•* Time and budget	*•* Time and budget *•* Support from the communication partner for marketing
**Evaluation success of movement app**	Successful: *•* Implementation by NSCs *•* Collaboration with gym teacher Unsuccessful: *•* Took a lot of time and energy *•* No communication packet *•* Start-up package missing	Successful: *•* Implementation by NSCs, area teams, and communication colleagues Unsuccessful: *•* City-wide approach *•* Lack of time during implementation	Successful: *•* Implementation by NSCs *•* Marketing Unsuccessful: *•* Driving force is missing in the organization

#### Time investment.

During implementation, municipalities emphasized the substantial and ongoing time and energy required to keep the app functional and relevant in daily practice. Unlike the adoption phase, where barriers centered on initial access or skepticism, here the challenge was sustaining use once the intervention was introduced. Implementers reported that updating content, engaging children continuously, and troubleshooting technical issues demanded skills and capacity that were often lacking. For one municipality, this workload prompted them to switch to an alternative, simpler app that required less maintenance but still met their goals. In other cases, the usability of the app for children became a bottleneck, as difficulties with navigation or technical bugs reduced participation.

#### Marketing.

Continuous marketing to end users was considered important for success. Municipalities that adopted strategies, such as collaborating with communication agencies or launching paid advertising campaigns saw higher levels of awareness and participation: *“So there were paid advertisements, targeting parents to make parents aware of this activity. We also had a website built, and a promotional video. All resources we put in place to raise awareness.”* (Municipality F). Large-scale, city-wide campaigns were perceived as less effective, while smaller-scale or neighborhood-focused efforts allowed for more tailored and impactful promotion.

Several materials that were needed during adoption and preparation remained important throughout implementation, particularly for local marketing, such as a communication or marketing toolkit with ready-to-use promotional materials, such as flyers, social media content, and example messages. In addition, examples of good practices from other municipalities were considered useful. For instance, one municipality adopted the use of a mascot after seeing this approach successfully applied elsewhere, illustrating how practical examples could support local promotion and engagement during implementation.

#### Monitoring and proven outcomes.

Municipalities stressed the importance of collecting both quantitative and qualitative evidence to support implementation. Usage statistics, such as downloads and missions completed, were combined with feedback from children, gym teachers, and colleagues. These data points were important for demonstrating the added value of gamification and securing ongoing funding. Municipalities reported positive outcomes such as improved motor skills, exposure to new sports, and engagement of previously inactive target groups as important measures to monitor. Yet, policymakers consistently emphasized the need for proven outcomes before committing to long-term implementation: *“You’re going to get questions of ‘what does it deliver?’ We have to be able to show that, otherwise policymakers will never support it.”* (Municipality E). The lack of long-term evidence was therefore seen as a barrier to embedding gamification structurally into municipal policy.

#### Support and leadership.

During implementation, the type and duration of political and organizational support proved important for sustaining momentum. Unlike in the adoption phase, where leadership primarily influenced whether pilots were initiated, here the focus shifted to maintaining resources and commitment once gamification had begun. Strong political backing from aldermen helped secure budget allocations and institutional visibility, while dedicated enthusiastic individuals, such as implementers or communication staff ensured continuity in day-to-day delivery. *“Look, at the end of the day, you can write visions and policy papers all you want, but if an alderman has a very strong desire for something [...] then the chances are pretty good that it happens.”* (Policymaker). However, this support was often tied to temporary funding schemes or short-term projects, limiting municipalities’ ability to embed gamification structurally. Where long-term leadership commitment was absent, interventions risked stagnation or discontinuation despite early enthusiasm.

#### Policy landscape.

Finally, the broader policy landscape influenced implementation trajectories. Changes in political leadership or shifting policy priorities posed threats to ongoing efforts in practice. For instance, when a new alderman entered with a different focus, gamification risked being sidelined. At the same time, municipalities noted that actively connecting gamification to emerging policy priorities helped maintain relevance and protect investments already made.

### Scaling: sustainment & stabilization phase

Three sub-themes were identified for the scaling phase (Table 8). Scaling was rarely achieved in practice. Most municipalities described themselves as still working toward scaling, reflecting uncertainty about how to secure gamification structurally within their policy and organizational systems: *“We are now in a phase where things are going okay. How are you going to secure this now and how are you going to make sure that this, that it’s something, to have a continuous existence within the municipality?”* (Municipality A).

**Table 8:** Identified facilitators and challenges in the scaling phase.

Sub-theme	Facilitators	Challenges
**Scaling out** (replication across municipalities)	*•* Company prioritized societal impact over profit in the short-term *•* Company emphasized geographic spread and user volume *•* Company executed entrepreneurial strategies (networking, free trials, business cases) *•* Engagement between company with researchers, health professionals, and policymakers	*•* Variation in municipal policies *•* Reliance on municipal adoption
**Scaling in** (integration within sport organizations)	*•* Close collaboration between policymakers and health professionals *•* Short communication lines and feedback loops	*•* Differences in adaptability innovation
**Scaling up** (embedding into policy and systems)	*•* Some municipalities embedded gamification structurally *•* Broad framing supporting budget and reduce administrative burdens *•* Integrating gamification into education, health, well-being, and economy domains *•* Alignment with the Gezond en Actief Leven Akkoord (GALA) *•* Allocated dedicated funds	*•* Gamification as a flexible tool for short-term goals rather than a long-term policy priority *•* Uncertainty about which policy domains to involve and how to coordinate *•* Short-term funding

In addition to these sub-themes, municipalities without gamification experiences (municipalities D-F) reflected more broadly on their innovation capacity to scale sport and movement innovations. Both health professionals and policymakers recognized improvements. However, clear divergences in perception emerged between health professionals and policymakers. For example, policymakers generally felt financial resources were insufficient, while health professionals considered them adequate. This difference may reflect the distinct roles of these stakeholders. Policymakers typically have greater insight into municipal budget constraints, competing policy priorities, and anticipated future financial pressures, which may make them more cautious about the long-term availability of resources. In contrast, health professionals may assess resource adequacy primarily from an operational perspective, focusing on the resources available for delivering and supporting the intervention in practice. Policymakers questioned whether favorable conditions for innovation were present, whereas health professionals agreed these conditions existed. Differences further appeared in views on embedding small-scale innovations into routine practice and on the adequacy of internal information exchange. These contrasts illustrate how internal differences within municipalities can influence the pathway from adoption toward scaling.

#### Scaling out (replication across municipalities).

The game company focused on outscaling the movement app to multiple municipalities. The company emphasized the importance of sustainability: *“We shouldn’t just throw new products out there. We need to ensure they are implemented into society first”*. Their strategy emphasized societal impact over profit, highlighting both geographic spread and user volume as essential for further development: *“So we thought, it would of course be great if the whole of the Netherlands could play the game, but for that you need some kind of national coverage. It really started from the municipalities, not to make money or profit, but more like, if at some point you have 100,000 children using it, then you have an impact and then you can also develop more easily”*. Entrepreneurial strategies such as networking, offering free trials, and building a business case supported this process, as did collaboration with researchers, health professionals, and policymakers.

#### Scaling in (integration within sport organizations).

Municipalities noted the importance of collaboration between policymakers and health professionals to integrate gamification into the work of implementers (e.g., sport organizations). Short communication lines and feedback loops supported translation of local practices into municipal priorities: *“There are very short lines of communication with the policy makers who are in charge of sports […]. It happens the other way around as well if things are going on in the field. So, interaction is essential.”* (Municipality C). Differences in adaptability were evident, with some teams embracing gamification more readily than others.

#### Scaling up (embedding into policies and systems).

Municipalities differed in how they positioned gamification in their policy frameworks. A minority (2/6) included gamification explicitly in their policy plans, either as part of a structured multi-year strategy or embedded within innovation agendas. This broad framing helped to secure budgets and reduce administrative hurdles. In contrast, most municipalities treated gamification mainly as a flexible tool for achieving short-term goals rather than as a long-term priority: *“The municipality gives us the assignment and as a sports company we can decide for ourselves how we fulfill those goals and that assignment.”* (Municipality C). Another factor shaping scaling up was the degree of integration across policy domains. Municipalities that positioned gamification within education, health, well-being, and economic strategies found it easier to mobilize support and resources. Alignment with the Dutch *Gezond en Actief Leven Akkoord* (Healthy and Active Living Agreement, GALA) was regarded as a promising framework for scaling gamification. GALA is a national agreement designed to strengthen prevention and promote healthy living by stimulating integrated collaboration across domains such as health, youth, and sport. It encourages municipalities to work more structurally with health professionals (e.g., NSCs) and to embed preventive activities within long-term local policy. This makes GALA a relevant policy framework for structurally positioning gamification within municipal practice, as it fosters collaboration across domains and encourages municipalities to employ NSCs more structurally. For example, gamification can simultaneously support sport goals (e.g., increasing physical activity participation), health goals (e.g., promoting healthy lifestyles and prevention), and youth goals (e.g., engaging children and adolescents through attractive activities). This shared objective can facilitate collaboration between NSCs, public health professionals, schools, and youth workers. Looking ahead, municipalities recognized the challenge of determining which policy domains should be involved and how coordination across them should be organized. In some cases, municipalities allocated funds specifically to facilitate structural implementation and scaling. However, financial constraints frequently limited scaling of successful pilots. Funding was often short-term and tied to temporary subsidies, restricting municipalities’ ability to sustain gamification efforts over time.

## Discussion

This study examined how municipalities adopt, implement, and scale gamified PA interventions, focusing on the case of a digital PA intervention (i.e., ‘Missie Master’ app) in Dutch municipalities. We show that a practical decision pathway for adoption, implementation, and a three-part scaling typology (scaling in, scaling out, scaling up) helped in translating gamification from pilots into policy and routine practice across municipal, professional, and industry stakeholders. In line with prior work, municipalities lacked clear decision frameworks, under-resourced implementation support, and struggled to move beyond pilots [24,26]. Three main lessons learned can be drawn relating to adoption, implementation, and scaling.

Adoption (i.e., exploration, adoption/preparation & emergence phase) of gamified PA interventions was shaped primarily by informal decision-making processes rather than systematic evaluation. Enthusiastic individuals, trust in product reliability, and exchange between municipalities determined whether adoption occurred. This reliance on individual enthusiasm reflects the absence of transparent, consistent frameworks to guide adoption. While effectiveness and cost-effectiveness are commonly cited [17,29], our results also highlighted the importance of combining qualitative and quantitative evidence at the point of adoption. Early evidence not only demonstrates immediate relevance but also lays the foundation for proving sustainability in later phases. Recruitment of municipalities further revealed a misalignment between companies’ fast-paced innovation cycles and municipalities’ slower bureaucratic timelines, making uptake difficult to coordinate [24,40]. Personalized outreach by the company itself or recommendations from trusted colleagues (e.g., health professionals, or policymakers) proved decisive, whereas generic communication was generally ignored. These findings suggest that adoption is not simply a matter of interest or capacity but also on how decisions are formally made within municipalities, including who is involved, which priorities are considered, and what evidence is required. Our decision guide (Table 5) provides a first step toward addressing this gap by clarifying the questions policymakers and health professionals consider, the evidence they require, and the roles each plays in assessing whether and how to adopt a gamified intervention. This can help companies to better align their communication with municipal needs and thereby increase the likelihood of successful scaling, instead of ‘reinventing the wheel’ of developing new products. These findings suggest that adoption (i.e., exploration, adoption/preparation, and emergence phases) should not be viewed as a stand-alone phase. During exploration, municipalities assessed the fit between the intervention and local needs, while adoption/preparation involved gathering evidence, engaging stakeholders, and building organizational commitment. These early activities shaped the emergence of gamification beyond isolated pilots and influenced subsequent implementation readiness and scaling opportunities.

Once adopted, implementation success (i.e., implementation & institutionalization phase) depended less on the intervention itself and more on municipal readiness to implement. Health professionals were central to implementing, but frequently reported insufficient organizational or political backing, echoing evidence that municipal infrastructure and readiness, rather than the quality of the innovation, determine outcomes [14,41]. Implementation was hindered by resource constraints, in terms of time and skills, keeping the app updated, and marketing efforts. Municipalities often lacked clear communication or marketing strategies, and monitoring templates to track both usage and perceived impact. Where such supports were missing, implementation became resource-intensive and reliant on individual improvisation. Political and organizational support was also fragile, often tied to temporary funding schemes, which limited the ability to sustain delivery beyond initial pilots. This highlights that implementation and institutionalization are closely connected. Although health professionals were able to deliver the intervention, broader organizational embedding depended on stable resources, clear communication structures, monitoring systems, and continued political support. These findings highlight that adoption decisions alone are insufficient. Structured pre-implementation planning is crucial [14,40–43]. Facilitated communities of practice could further strengthen intermunicipal exchange, as municipalities valued opportunities for peer learning but did not actively use the online platform provided. Without such infrastructure, implementation (i.e., institutionalization) risks remaining ad hoc and short-term rather than a stepping stone toward scaling [41].

Scaling (i.e., sustainment & stabilization phase) was rarely achieved, suggesting that gamified PA interventions are still in its infancy, with a lack of proven effects [14], or a long timeline in scaling [22]. Movement between piloting, partial implementation, and scaling supports the view that the process of moving from one phase is iterative, not linear. Consistent with scaling-out [21] and scale-up [26], effective scaling requires readiness, adaptation, and empirical justification in new settings rather than simple replication. Embedding scaling thinking at the outset, designing pilots with implementation and scaling in mind, reduces the risk of cycling through short-term projects. Across phases, leadership and close policy–practice links were a recurrent enabler. Short communication lines and visible enthusiastic individuals accelerated progress, while unclear responsibilities between policymakers and health professionals and variation in municipal policies and expectations slowed it. Operationalizing scaling in/out/up proved useful in this project. Scaling in (integration within sport organizations) clarified the organizational changes and short feedback loops needed between policymakers and implementers [14,41]. Scaling out (replication across municipalities) highlighted the dependencies of companies on local policy fit and the value of endorsements by other municipalities. Scaling up (policy/system embedding) surfaced the importance of broad framing (e.g., innovation agendas), cross-domain integration, and stable funding (e.g., alignment with GALA). The overlap between adoption, implementation, and scaling observed in this study reinforces the value of combining EPIS and the Validated Scaling Instrument. Decisions made during exploration and adoption influenced implementation readiness, while implementation experiences shaped opportunities for sustainment and stabilization.

### Recommendations

Based on our findings, several actions appear crucial to strengthen the adoption, implementation, and scaling of gamified PA interventions in municipal contexts (Box 1). Pilot projects are an important precondition for gaining political and organizational support. Our study confirmed that visible outcomes were important in moving beyond initial skepticism. This reflects earlier findings that legitimacy and trust in effectiveness must be established before municipalities are willing to take the next step, and sharing best practices or providing an overview of available tools might help [14,41].

Municipalities may need to consider appointing a designated coordinator to oversee the adoption process and ensure continuity across departments and projects [14,41]. At the same time, the presence of enthusiastic individuals and supportive leadership is essential to build momentum and secure long-term support. To ensure long-term embedment, clear assignments and structural support are needed to prevent dependence on temporary enthusiastic individuals and to build lasting capacity [14].

Once these structures are established, municipalities should work toward integration across policy domains such as education, youth, and sports, thereby creating the conditions for both implementation and scaling. Besides, exchanging within and beyond municipalities seemed useful, which might be facilitated by a national network [14,41]. Such cross-over exchange can help to bundle efforts share best practices, and coordinate pilot testing, thereby providing policymakers with visible evidence of effectiveness and legitimacy to justify broader implementation and scaling.

Involving a variety of stakeholders, including health professionals and policymakers early in the development of gamified health interventions is also key. This ensures that interventions are aligned with municipal priorities, professional practice, and user needs. Municipalities and companies should engage in early-stage co-development, ideally supported by national frameworks or innovation partnerships that can provide legitimacy, shared resources, and guidance for aligning local priorities with technological development. Alongside this, structured and transparent evaluation benchmarks are needed that go beyond usage statistics (e.g., downloads, mission participation) to also capture policy integration (e.g., embedding in budgets, strategic plans).

Future research should build on these insights by focusing on longitudinal approaches to scaling [30], strategies to ensure successful implementation before scaling [14], and stronger facilitation of intermunicipal collaboration [44]. Addressing these challenges will be important for advancing the real-world adoption, sustained implementation, and scaling of gamified health interventions.

**Box 1. Recommendations for adopting, implementing, and scaling gamified PA interventions.**

**First actions:**

1. Pilot projects with visible outcomes
2. Designated coordinators
3. Supportive leadership and enthusiastic individuals

**Next actions:**

1. Cross-domain integration
2. Network exchange
3. Early-stage co-development in future intervention developments
4. Structured evaluation

### Strengths and limitations

This study has several strengths and limitations that should be considered. A key strength lies in its focus on the municipal scaling process of gamified PA interventions, an area that remains underexplored. By applying and validating scaling frameworks from other domains [30,32], this project extended its use to the health sector, contributing to the broader field of gamification and public health policy. The study also examined public innovation capacity at two time points, going beyond previous research [36] by highlighting how municipal actors perceive their capacity for innovation and change over time. Importantly, the use of the scaling instrument not only provided insights but also functioned as a self-assessment and awareness tool, initiating conversations between policymakers and health professionals. Including both groups in the process allowed the study to capture multiple perspectives, reflecting the interdependent roles needed to support scaling efforts.

At the same time, several limitations should be acknowledged. First, due to time constraints, the project was limited to the early experimental phase of scaling. Although scaling is inherently iterative, the second workshop could not return to the scaling instrument for further refinement, which may have slowed progress toward implementation and scaling. Second, the innovation capacity questionnaire proved challenging for health professionals with limited knowledge of municipal processes, which made certain questions difficult to answer [36]. This highlighted the subjectivity inherent in assessing public innovation capacity and the need for further longitudinal research to capture scaling dynamics across phases more fully. Third, the study involved a relatively small sample size, including only a few municipalities, a limited number of participants within municipalities, and only one company. Although the participating municipalities varied in size and experience with gamification, they may not fully represent the diversity of the broader Dutch municipal landscape. Municipalities differ in their resources, governance structures, policy priorities, and levels of experience with innovation, which may influence adoption, implementation, and scaling processes. Consequently, the barriers, facilitators, and decision-making pathways identified in this study may not be equally applicable across all Dutch municipalities. Furthermore, the findings should be interpreted within the specific Dutch policy context in which the study took place. Finally, while the project emphasized the importance of including both policymakers and health professionals, a recurring challenge was the lack of clear role distribution between these groups. This challenge was already evident during adoption, where municipalities reported uncertainty about who should take responsibility for adopting the intervention. To address this, municipalities may benefit from explicitly defining ownership and responsibilities early in the adoption process. For example, policymakers may lead strategic decision-making, policy alignment, and funding, while health professionals may lead operational implementation, participant engagement, and feedback from practice. This may facilitate coordination and support subsequent implementation and scaling efforts, echoing earlier findings that role clarity is essential for collaborative health innovation [14].

### Conclusions

This study provides valuable insights into the adoption, implementation, and scaling of gamified PA interventions. Our findings show that fragmented adoption, weak implementation infrastructures, and uncertain scaling strategies currently limit sustainable integration in municipal policy and practice. Moving forward, three priorities are essential: 1) establishing transparent decision-making frameworks to guide adoption, 2) investing in structured early-stage preparation to build implementation capacity, and 3) reframing scaling as an iterative, long-term process embedded from the start of intervention development. Addressing these priorities requires closer collaboration between policymakers, health professionals, and companies. Only through such coordinated efforts, health innovation may move beyond pilots and become a sustainable part of municipal health policy and practice.

## Supporting information

S1 Text Description of mission master. (DOCX)

S2 Text Inspiration cards phase 2. (DOCX)

S3 Text Workshop material. (DOCX)

S4 Text Observation guide. (DOCX)

S5 Text Coding frame. (DOCX)

## Abbreviations

EPIS exploration: adoption/preparation, implementation, and sustainment
KcSB: Knowledge Centre for Sport & Physical Activity
NSC: neighborhood sport coach
PA: physical activity.
