# Supplementary material for: Decision-making pathways for adopting, implementing, and scaling gamified physical activity interventions in municipal policy and practice in the Netherlands
Source: PLOS Digit Health. 2026 Oct 26;5(10):e0001792. doi: 10.1371/journal.pdig.0001792 (PMC13704860; doi:10.1371/journal.pdig.0001792)
Supplement: Supplementary file 1 [file pdig.0001792.s001.docx]

## S1 Description of Mission Master

| Name | Mission Master |
| --- | --- |
| Developer | [removed] |
| Country | The Netherlands |
| Health topic | Primarily physical activity; adaptable to other health topics |
| Targeted age group | Children aged 8–12 years |
| Implementers | Neighborhood sport coaches, sport coaches, teachers |
| Technology mode | Mobile app (iOS, Android); online and offline functionality |
| Intervention content | Customizable “missions” created by implementers via backend, including:  • Physical activity missions (e.g., GPS scavenger hunts, offline code collection)  • Knowledge questions  • Lifestyle missions (e.g., fruit and vegetable consumption)  • Promotion of local sports clubs or activities  • Mini-games for additional engagement  Children earn medals and avatar outfits by completing missions. |
| Mode of use | • Active mode: customizable missions by implementers  • Passive mode: mini-games and avatar customization |
| Costs | Free to download (App Store, Google Play). App can be installed on parent/guardian device; missions can be completed without carrying a phone (codes entered later). |
| Usability/content testing | Co-designed and tested with children and neighborhood sport coaches |
| Contextual adaptability | Municipality owns the app account (paid version) and can tailor content to local needs and policy goals |
| Story | Six spies (Evi, Kai, Jesse, Alex, Omar, Dane) solve mysteries using gadgets, involving both physical and cognitive challenges |
| Limitations for delivery at scale | Designed for municipal adaptation; contextual factors influencing scalability examined in this study. |
| Data security | GDPR compliant; no personal data collected. Only aggregated backend data (e.g., downloads, mission completion) are traceable. |
| Link | [removed]  [removed] |

*Impression of Mission Masters backend (for coaches/implementers)*

|  | [picture removed] |
| --- | --- |
|  |  |

*Mission Master characters are visible both online and offline in [removed], connecting offline physical exercises with online rewards*

|  | [picture removed] |
| --- | --- |
