# Supplementary material for: Decision-making pathways for adopting, implementing, and scaling gamified physical activity interventions in municipal policy and practice in the Netherlands
Source: PLOS Digit Health. 2026 Oct 26;5(10):e0001792. doi: 10.1371/journal.pdig.0001792 (PMC13704860; doi:10.1371/journal.pdig.0001792)
Supplement: Supplementary file 2 [file pdig.0001792.s002.docx]

## S2 Inspiration cards phase 2

**Facilitators**

Importance of entrepreneurial skills within the municipality or intermediary organization

Vision and motivation of the alderman

Financial support

Support within the municipality from various departments

Collaboration with other municipalities

Network within/outside the municipality

Trust in the company or product

Few entrepreneurial skills within the municipality

Lack of enthusiastic policymakers

**Barriers**

Little support from the alderman

Stigmas around gamification

Limited financial support

No long-term support over multiple years

Changing political landscape

Barriers to implementation within intermediary organizations

Marketing to end users

Keeping EGG products up to date

Implementation: uncertainty about use or application

Little room to experiment (e.g., Living Lab)

**Strategies**

Creating support by, for example, sharing a personal story and vision

Investing money as a sign of commitment

Policy integration (SPUK/GALA, other policy plans? how to link to other domains?)

Product with high user-friendliness

What factors/conditions should a company offer?

How important is product ownership? – shared investment and responsibility

Free trials of products

"Just get started"

Include in the training/education of neighborhood sports coaches

Favorable entrepreneurial climate

**translated by ChatGPT*
