# Supplementary material for: Decision-making pathways for adopting, implementing, and scaling gamified physical activity interventions in municipal policy and practice in the Netherlands
Source: PLOS Digit Health. 2026 Oct 26;5(10):e0001792. doi: 10.1371/journal.pdig.0001792 (PMC13704860; doi:10.1371/journal.pdig.0001792)
Supplement: Supplementary file 3 [file pdig.0001792.s003.docx]

## S3 Workshop material

### Public Innovation Capacity questionnaire

| **Statements for self-assessment** | **Strongly disagree** | **Disagree** | **Neutral** | **Agree** | **Strongly agree** |
| --- | --- | --- | --- | --- | --- |
| Municipal employees with ideas about physical activity and sport innovation can easily find the right people within the municipality to realize these ideas. | ☐ | ☐ | ☐ | ☐ | ☐ |
| The drivers of physical activity and sport innovation in the municipality succeed in involving companies, scientists, and citizens in the development of ideas. | ☐ | ☐ | ☐ | ☐ | ☐ |
| The municipality has a strong network of companies, scientists, and citizens who are structurally engaged in physical activity and sport innovation. | ☐ | ☐ | ☐ | ☐ | ☐ |
| The drivers of physical activity and sport innovation in the municipality succeed in encouraging colleagues within the municipality to develop ideas in this field. | ☐ | ☐ | ☐ | ☐ | ☐ |
| The municipality has a lively network of employees with an interest in physical activity and sport innovation. | ☐ | ☐ | ☐ | ☐ | ☐ |
| A company, scientist, or citizen with good ideas in this field can easily find the right people within the municipality to work them out together. | ☐ | ☐ | ☐ | ☐ | ☐ |
| The municipality is successful in setting up experiments in the field of physical activity and sport innovation. | ☐ | ☐ | ☐ | ☐ | ☐ |
| The political leadership (Municipal Council and Executive Board) supports experiments in this field. | ☐ | ☐ | ☐ | ☐ | ☐ |
| The senior civil service supports experiments in this field. | ☐ | ☐ | ☐ | ☐ | ☐ |
| The municipality allocates sufficient financial resources for experiments in this field. | ☐ | ☐ | ☐ | ☐ | ☐ |
| The municipality involves, when necessary, other governments, companies, and civil society organizations in experiments in this field. | ☐ | ☐ | ☐ | ☐ | ☐ |
| The municipality is successful in scaling up experiments in this field. | ☐ | ☐ | ☐ | ☐ | ☐ |
| The municipality incorporates proven successful small-scale innovations into regular working methods. | ☐ | ☐ | ☐ | ☐ | ☐ |
| The municipality adequately evaluates experiments in this field. | ☐ | ☐ | ☐ | ☐ | ☐ |
| The municipality transforms experimental collaborations with other stakeholders into structural collaborations. | ☐ | ☐ | ☐ | ☐ | ☐ |
| The municipality succeeds in identifying risks, drawbacks, and tensions in this field. | ☐ | ☐ | ☐ | ☐ | ☐ |
| The municipality initiates public debate on risks, drawbacks, and tensions in this field and how to address them. | ☐ | ☐ | ☐ | ☐ | ☐ |
| The municipality is successful in resolving conflicts in this field. | ☐ | ☐ | ☐ | ☐ | ☐ |
| Ethical aspects of physical activity and sport innovation are adequately discussed within the municipality. | ☐ | ☐ | ☐ | ☐ | ☐ |
| The municipality allocates structural financial resources for physical activity and sport innovation. | ☐ | ☐ | ☐ | ☐ | ☐ |
| There is good information exchange on physical activity and sport innovation between all parties within the municipality. | ☐ | ☐ | ☐ | ☐ | ☐ |
| The municipality has a culture oriented toward physical activity and sport innovation. | ☐ | ☐ | ☐ | ☐ | ☐ |
| The municipality creates good preconditions (training, resources, information-sharing) for physical activity and sport innovation. | ☐ | ☐ | ☐ | ☐ | ☐ |
| The municipality has a clear vision on physical activity and sport innovation. | ☐ | ☐ | ☐ | ☐ | ☐ |
| The political leadership (Municipal Council and Executive Board) is willing to allocate budgetary resources for physical activity and sport innovation. | ☐ | ☐ | ☐ | ☐ | ☐ |

### Public Innovation Capacity reflection

**Part B: What is the capacity of each municipality to innovate in the field of physical activity and sport innovation?**

*Physical activity and sport innovation = the development and/or introduction of new products, services, and activities aimed at promoting sport and physical activity.*

*Based on the questionnaire that everyone completed individually:*

| How do you describe physical activity and sport innovations? What falls under this definition? |  |
| --- | --- |
| Do you see differences in the answers? Why or why not? |  |
| If so, what do you think caused these differences? For example, differences in perception or urgency? Differences in function? |  |
| What are strengths within the municipality? |  |
| What are weaknesses within the municipality? |  |

### Implementation plan

| **Main Question** | **Answer** |
| --- | --- |
| Has your organization/municipality set goals regarding the implementation of *Missie Master*? |  |
| If yes, what are the objectives? |  |
| How does *Missie Master* relate to other/comparable interventions in your municipality? |  |
| Will *Missie Master* replace or complement an existing program? If yes, how? |  |
| What advantages do you see in using *Missie Master*? |  |
| What disadvantages do you see in using *Missie Master*? |  |
| Who will lead the implementation of *Missie Master* in your municipality? (e.g., a health professional or team) |  |
| Who else will be involved in leading the implementation? (e.g., colleagues) |  |
| What type of support or actions can you expect from leaders in your organization to make the implementation successful? (e.g., providing sufficient time, space, additional resources such as marketing materials) |  |
| Will someone (or a team) outside your organization help you implement *Missie Master*? (e.g., teachers in schools, coaches in sports clubs) If yes, who? What role? What activities are planned? |  |
| Are there key influential people who want to participate in this implementation? If yes, who? |  |
| How will *Missie Master* be integrated into the daily work of the health professional/teacher? (e.g., during weekly programming, larger events, or holiday activities) |  |
| Which target group will you focus on? |  |
| What is your marketing/communication strategy to raise awareness of *Missie Master*? (e.g., online/offline material, locations such as schools, clubs, or youth centers) |  |
| Do you expect to have sufficient resources to execute and manage *Missie Master*? Which resources do you consider important to include in the planning? |  |
| What type of information do you plan to collect during the implementation of *Missie Master*? What will you monitor? How will this information be used? |  |
| How will you assess progress toward the implementation objectives? |  |
| Will feedback be collected from those involved? (e.g., the target group, or individuals who supported implementation) |  |

### Implementation Evaluation

| **Original CFIR Item** | **Title** | **Main Question** | **Sub-questions / Details** |
| --- | --- | --- | --- |
| **Goals/Feedback** | Goals | Were the goals adjusted during the past months? If yes, how? Were the goals achieved? |  |
| **Relative Advantage** | Comparable interventions | Did *Missie Master* replace or complement an existing program? If yes, how? What advantages did you see in using *Missie Master*? What disadvantages did you see in using *Missie Master*? |  |
| **Formally appointed internal implementation leaders** | Responsibility | Who led the implementation of *Missie Master* in your municipality? Was this experienced as successful? (e.g., a health professional or team) |  |
| **Formally appointed internal implementation leaders** | Shared responsibility | Who else was involved in leading the implementation? Was this experienced as successful? (e.g., colleagues) |  |
| **Leadership engagement** | Internal organizational support | What type of support or actions did you receive from leaders in your organization to make the implementation of *Missie Master* successful? Was this experienced as successful? (e.g., providing sufficient time, space, or extra resources such as marketing materials) |  |
| **External Change Agents** | External organizational support | Did someone (or a team) outside your organization help implement *Missie Master*? If yes, who? What role? What activities were planned? Was this experienced as successful? (e.g., teachers in schools, coaches in sports clubs) |  |
| **Opinion leaders** | Influential persons | Were there important influential persons who wanted to participate in the implementation? If yes, who? Was this experienced as successful? |  |
| **Compatibility** | Integration in professional role | How was *Missie Master* integrated into the daily work of the health professional/teacher? Was this experienced as feasible? (e.g., during weekly programming or larger events/holiday activities) |  |
| **Key stakeholders** | Target group | Which target group did you focus on? |  |
| **Key stakeholders** | Marketing | What was your marketing/communication strategy to raise awareness of *Missie Master*? Was this considered successful within your municipality? Why/why not? (e.g., online/offline material, locations such as schools, clubs, youth centers) |  |
| **Available Resources** | Resources | Did you have sufficient resources to implement and manage *Missie Master*? Was this experienced as sufficient? What else would have been needed? |  |
| **Reflecting & Evaluating** | Monitoring | What type of information did you collect during the implementation of *Missie Master*? What did you monitor? How did you use this information further? |  |
| **Reflecting & Evaluating** | Assessing progress | How did you assess the progress of the implementation objectives? |  |
| **Reflecting & Evaluating** | Feedback | Was feedback obtained from those involved? If yes, what was the feedback? (e.g., from the target group, or from persons who supported implementation) |  |
| **Reflecting & Evaluating** | Challenges | What were the main challenges you encountered during the implementation of *Missie Master*? |  |
| **Reflecting & Evaluating** | Lessons learned | What would you have done differently in hindsight? |  |
| **Reflecting & Evaluating** | Future plans | Is your municipality planning to offer *Missie Master* again in the future? Why/why not? Is your municipality planning to offer another form of gaming or gamification in the future? Why/why not? |  |

### Validated Scaling Instrument

Municipality:

Value of Gamification:

In which phase are you? What do you exactly do?

Which challenges do we currently face?

Which strategies can we apply?

Which challenges do we expect?

Which strategies can we apply?
