# Supplementary material for: Decision-making pathways for adopting, implementing, and scaling gamified physical activity interventions in municipal policy and practice in the Netherlands
Source: PLOS Digit Health. 2026 Oct 26;5(10):e0001792. doi: 10.1371/journal.pdig.0001792 (PMC13704860; doi:10.1371/journal.pdig.0001792)
Supplement: Supplementary file 4 [file pdig.0001792.s004.docx]

## S4 Observation Guide

**Observation Form “How innovative is our municipality in the field of physical activity and sports innovation?” (plenary)**

| **Question 1** |  |
| --- | --- |
| What do participants have to add to the definition of physical activity and sports innovation? |  |
| Do participants agree with the definition of physical activity and sports innovation? Why or why not? |  |
| **Question 2** |  |
| How does the process of standing on the red line work (e.g., hesitating? looking at participants from their own municipality? or making a firm decision?) |  |
| Where do municipalities position themselves on the red line? |  |
| Where do policymakers versus Health professionals (within the same municipality) position themselves on the red line? |  |
| What arguments do policymakers versus Health professionals (across municipalities) provide? | Policymakers:            Health professionals: |
| Do we see differences/similarities between the arguments? If yes, which ones? |  |
| Other remarks |  |

**Observation Form “What is hypothetically needed to integrate physical activity apps into policy?”**

| **Question 1** |  |
| --- | --- |
| How does the process of standing on the red line work (e.g., hesitating? looking at participants from their own municipalities? or making a firm decision?) |  |
| Where do municipalities position themselves on the red line? |  |
| Where do policymakers versus health professionals (within the same municipality) position themselves on the red line? (e.g., do they agree/disagree?) |  |
| What arguments do policymakers versus health professionals (across municipalities) provide? | Policymakers:            Health professionals: |
| Do we see differences/similarities between the arguments? |  |
| **Question 2** |  |
| What barriers are mentioned? |  |
| Do municipalities agree or disagree with each other? Why or why not? |  |
| **Question 3** |  |
| What strategies are mentioned? |  |
| Do municipalities agree or disagree with each other? Why or why not? |  |
| **Question 4** |  |
| What outcome effects are mentioned? (e.g., scientific, SROI, etc.) |  |
| Do participants agree within and across municipalities? |  |
| **Other remarks** |  |
