# Supplementary material for: Decision-making pathways for adopting, implementing, and scaling gamified physical activity interventions in municipal policy and practice in the Netherlands
Source: PLOS Digit Health. 2026 Oct 26;5(10):e0001792. doi: 10.1371/journal.pdig.0001792 (PMC13704860; doi:10.1371/journal.pdig.0001792)
Supplement: Supplementary file 5 [file pdig.0001792.s005.docx]

## S5 Coding frame

| **Factors** | **Construct** | **Definition (underlined: sub codes)** |
| --- | --- | --- |
| **Framework for Scaling Social and Sustainable Initiatives** | | |
| Actor characteristics | Willingness - ambition | Social bricoleur: focuses on the local needs and will hardly touch the existing institutional context  Social constructionist: aims to replicate an innovation to different geographical areas  Social engineer: wants to break the (international) social system radically and focuses on the political agenda |
|  | Willingness – desire for control | Actor's wish to control  Consequently, a formal structure is chosen by actors who want to control, while open structures speed up scaling |
|  | Willingness - logic | Economic logic  Social logic  Economic and social logic |
|  | Ability – entrepreneurial skills | Skills to get political or financial support, to build networks, to hire professional staff, to communicate with stakeholders, to work efficiently, to build a proper business case and to develop marketing strategies. |
|  | Ability - leadership | Managing the internal organization and dealing with external circumstances |
|  | Ability - dependency | Dependency on money, knowledge or network |
| Pathway | Outscaling | Geographical level  Focuses on increasing numbers, from local to international or from small to big networks  (e.g., distribution of services, (financial) growth, differentiation, diversification and market development) |
|  | Upscaling | Policy level  Focuses on influence on local to international political agendas, public discourses or legislation  (e.g., influencing policy making, creating awareness, positive entrepreneurial environment, institutional context and inclusive policy making) |
|  | Open structure | The way in which initiatives organize their activities: open |
|  | Closed structure | The way in which initiatives organize their activities: closed |
| Impact | Volume | Size, larger reach e.g. within one municipality |
|  | Geo. distribution | Larger reach e.g. outside one municipality, distribution to other municipalities |
|  | System change | Changing an entire system, e.g. including economic, education and sport domains to reach impact |
| Institutional factors | Rules | Governments design and implement rules and actors can also influence these rules. Space for experimentation, flexible rules, a favorable business environment and political will are crucial for scaling local initiatives. |
|  | Support | Facilitating and encouraging role that government could have in supporting local initiatives. E.g., by train-the trainer, by connecting local initiatives with standards on higher levels or by co-management or workshops. |
| **The validated scaling instrument** | | |
| Phase of scaling process | Emergence | Making preparations to take the innovation out of its protective environment and apply it more widely in society or the organization |
|  | Institutionalization | Applying the innovation more widely in society or the organization for the first time |
|  | Stabilization | The innovation is already applied and securing it in existing or newly created structures |
| **Designing a Scaling Instrument for Public Sector Innovations** | | |
| Barriers | Stakeholder | Interaction  e.g. challenge to create support (e.g. games might have a negative image or are unknown, hard to prove the added value of apps), interact with third parties, differences between expectations and reality (e.g. overpromised solution) |
|  | Structural | Organizational and contextual  e.g. geographic differences in new structures, existing structures stagnate the scaling process, problems within the government |
|  | Execution | Innovation  e.g. insecurities stagnate the scaling process, practical barriers in implementation, the innovation has negative consequences (e.g. increased work load/ pressure) |
| Strategies |  | Concrete actions that are applied to overcome barriers |
| **Instrument for measuring public innovation capacity** | | |
| Capacity to innovate | Mobilizing | Connecting to other actors through boundary spanning activities.  E.g. finding suitable organizations and actors |
|  | Experimenting | Flexibility to act against or in the absence of formal procedures  E.g. (financial) support to experiment |
|  | Institutionalizing | Innovation is connected to the broader institutional environment  E.g. imbedding in structures |
|  | Balancing | Deliberately weighing the different interests and values involved in the process of public innovation and choosing the option that contributes most to societal desirable outcomes  E.g. Risks and conflicts are discussed |
|  | Coordinating | Need to manage the innovation system; managing connections, interfaces and relations between the various functions in the innovation system  E.g. Vision by policy makers, exchange, training |
| **Interview-related codes** | | |
| Municipality vs. Company | Municipality |  |
|  | Company |  |
| Collaboration municipality and intermediary organization | Municipality | Relationship between a municipality and intermediary organization; perspective from municipality |
|  | Intermediary organization | Relationship between a municipality and intermediary organization; perspective from intermediary organization that execute the sport agreement of the municipality, e.g. sport club, foundation, sport company that e.g. hire neighborhood sport coaches |
| Other municipalities |  | All municipalities in the Netherlands, other than the interviewed municipality |
| Implementation | Facilitating factors | e.g. capacity to implement games, alignment with needs of target group |
|  | Hindering factors | e.g. lack of exchange with colleagues, financial resources |
| EGG | E-sports | Professional play of games & all definitions that interviewees use to explain e-sports |
|  | Gaming | Using games to promote physical activity, yet, to play these games no physical activity is required  e.g. offering FIFA at a sports club |
|  | Gamification | Applying game elements to the real-life context to promote physical activity  e.g. movement walls |
|  | Movement app | Use of a digital application to promote physical activity  e.g. missie master |
| Policy plan |  | Policy plans linked to sport, innovation or other that eventually facilitate or hinder the implementation of movement apps |
| Future | Tips | Tips for other municipalities |
|  | Plans | Plans for own municipality |
|  | Requirements | What is needed by others (e.g. municipality) to facilitate the sustainable implementation |
| **Inductive codes** | | |
| Evaluation | Monitoring | What are the effects of the app |
| Only sub codes |  | To make some codes more specific |
