# Supplementary material for: Decision-making pathways for adopting, implementing, and scaling gamified physical activity interventions in municipal policy and practice in the Netherlands
Source: PLOS Digit Health. 2026 Oct 26;5(10):e0001792. doi: 10.1371/journal.pdig.0001792 (PMC13704860; doi:10.1371/journal.pdig.0001792)
Supplement: Supplementary file 6 [file pdig.0001792.s007.docx]

**Additional Editor Comments (if provided):**

The reviewers are quite positive about your manuscript. Very minor feedback has been provided which the authors are encouraged to address.

Dear Editor,

We thank you for the positive evaluation of our manuscript entitled "*Decision-making pathways for adopting, implementing, and scaling gamified physical activity interventions in municipal policy and practice in the Netherlands*" and for the opportunity to revise it. We appreciate the constructive feedback provided by the reviewers. We have carefully considered all comments and suggestions and have revised the manuscript accordingly. The changes made are detailed in our point-by-point response below. We believe these revisions have strengthened the clarity, practical relevance, and overall quality of the manuscript. We would like to thank you for your comments with regard to the formatting, and have adapted declaration of interest and financial disclosure according to the journal's guideline. Below you can find a detailed overview of all comments by the Editor and Reviewers, our response, and related manuscript adaptations. We resubmitted an unmarked version of our revised manuscript (‘Manuscript’) version and a marked-up version (‘Revised Manuscript with Track Changes’) for your convenience.

Sincerely,

Maarten van Bottenburg and co-authors

**Editors**

**Journal Requirements:**

1. Please amend your detailed *Financial Disclosure statement*. This is published with the article. It must therefore be completed in full sentences and contain the exact wording you wish to be published.

i. Please clarify all sources of financial support for your study. List the grants, grant numbers, and organizations that funded your study, including funding received from your institution. Please note that suppliers of material support, including research materials, should be recognized in the Acknowledgements section rather than in the Financial Disclosure. 
ii. State the initials, alongside each funding source, of each author to receive each grant. For example: "This work was supported by the National Institutes of Health (####### to AM; ###### to CJ) and the National Science Foundation (###### to AM)."
iii. State what role the funders took in the study. If the funders had no role in your study, please state: “The funders had no role in study design, data collection and analysis, decision to publish, or preparation of the manuscript.”
iv. If any authors received a salary from any of your funders, please state which authors and which funders.

2. Please send a completed *'Competing Interests' statement*, including any COIs declared by your co-authors. If you have no competing interests to declare, please state "The authors have declared that no competing interests exist". Otherwise please declare all competing interests beginning with the statement "I have read the journal's policy and the authors of this manuscript have the following competing interests:"

3. We note that your *Data Availability Statement* is currently as follows: All materials underlying this article are available in Supporting Information.
 
Please confirm at this time whether or not your submission contains all raw data required to replicate the results of your study. Authors must share the “minimal data set” for their submission. PLOS defines the minimal data set to consist of the data required to replicate all study findings reported in the article, as well as related metadata and methods (<https://journals.plos.org/plosone/s/data-availability#loc-minimal-data-set-definition>).
 
 For example, authors should submit the following data:
 
 - The values behind the means, standard deviations and other measures reported;
 - The values used to build graphs;
 - The points extracted from images for analysis.
 
 Authors do not need to submit their entire data set if only a portion of the data was used in the reported study.
 
 If your submission does not contain these data, please either upload them as Supporting Information files or deposit them to a stable, public repository and provide us with the relevant URLs, DOIs, or accession numbers. For a list of recommended repositories, please see <https://journals.plos.org/plosone/s/recommended-repositories>.
 
 If there are ethical or legal restrictions on sharing a de-identified data set, please explain them in detail (e.g., data contain potentially sensitive information, data are owned by a third-party organization, etc.) and who has imposed them (e.g., an ethics committee). Please also provide contact information for a data access committee, ethics committee, or other institutional body to which data requests may be sent. If data are owned by a third party, please indicate how others may request data access.

4. In the online submission form, you indicated that “No additional data are available in order to protect participant privacy. Additional information can be obtained from the corresponding author upon reasonable request, while ensuring participant privacy is protected.”.

All PLOS journals now require all data underlying the findings described in their manuscript to be freely available to other researchers, either 
1. In a public repository, 
2. Within the manuscript itself, or 
3. Uploaded as supplementary information.

This policy applies to all data except where public deposition would breach compliance with the protocol approved by your research ethics board. If your data cannot be made publicly available for ethical or legal reasons (e.g., public availability would compromise patient privacy), please explain your reasons by return email and your exemption request will be escalated to the editor for approval. Your exemption request will be handled independently and will not hold up the peer review process, but will need to be resolved should your manuscript be accepted for publication. One of the Editorial team will then be in touch if there are any issues.

If the reviewer comments include a recommendation to cite specific previously published works, please review and evaluate these publications to determine whether they are relevant and should be cited. There is no requirement to cite these works unless the editor has indicated otherwise. 

**Reviewer #1**

**R1-C1:**

The authors provide a detailed review of their experience implementing a gamified physical activity intervention in various Netherlands municipalities. The data provides insight into enablers/barriers of implementation for these applications and within these settings. I suggest that the article be accepted after the following minor revisions are addressed.

**Response:**

We thank the reviewer for their positive assessment of our manuscript and for recognizing the contribution of our study in providing insights into the implementation of a gamified physical activity intervention across Dutch municipalities. We appreciate the constructive feedback and have carefully addressed all comments and suggestions. Below, we provide a detailed response to each point and indicate the corresponding revisions made to the manuscript.

**R1-C2:**

- The introduction introduces necessary concepts/frameworks, but consider organising this section with subheadings for the different concepts

**Response:**

In response to the reviewer’s comment, we have added subheadings to the Introduction to better structure the text according to the three key challenges addressed in this article: adoption challenges, implementation challenges, and scaling challenges. Further we created extra structure by splitting the paragraphs describing the concepts of scaling; i.e. out, up, deep, and beyond. And we aligned the adoption, implementation, and scaling phases from both frameworks and reflected on each in the discussion.

We thank the reviewer for this helpful suggestion. To improve readability and structure, we have added subheadings throughout the Introduction to organize the text around the three key challenges addressed in this study: adoption, implementation, and scaling of gamified physical activity interventions. We also introduced additional subheadings in the Theoretical Underpinning section to align the corresponding phases of the EPIS framework (exploration/adoption-preparation, implementation, sustainment) with those of the Validated Scaling Instrument (emergence, institutionalization, stabilization). Furthermore, we improved the structure of the scaling section by separating and clarifying the concepts of scaling in, scaling out, and scaling up. Finally, we revised the Discussion section to reflect this alignment between EPIS and the Validated Scaling Instrument, highlighting how the findings relate to the overlapping phases of exploration, adoption/preparation and emergence; implementation and institutionalization; and sustainment and stabilization.

**Adaptations to the manuscript**

Page 9; Line 186: Adoption: exploration, adoption/preparation & emergence phase

Page 10; Line 199: Implementation: implementation & institutionalization phase

Page 10; Line 207: Scaling: sustainment & stabilization phase

Page 6; Line 120: In contrast, the public sector innovation literature categorizes scaling into five distinct dimensions, each addressing different aspects of broadening the impact of innovations: scaling out, scaling up, scaling deep, and scaling beyond.

Page 35; Line 579: Adoption (i.e., exploration, adoption/preparation & emergence phase) of gamified PA interventions was shaped primarily by informal decision-making processes rather than systematic evaluation.

Page 35; Line 597: These findings suggest that adoption (i.e., exploration, adoption/preparation, and emergence phases) should not be viewed as a stand-alone phase. During exploration, municipalities assessed the fit between the intervention and local needs, while adoption/preparation involved gathering evidence, engaging stakeholders, and building organizational commitment. These early activities shaped the emergence of gamification beyond isolated pilots and influenced subsequent implementation readiness and scaling opportunities.

Page 36; Line 603: Once adopted, implementation success (i.e., implementation & institutionalization phase) depended less on the intervention itself and more on municipal readiness to implement.

Page 36; Line 612: This highlights that implementation and institutionalization are closely connected. Although health professionals were able to deliver the intervention, broader organizational embedding depended on stable resources, clear communication structures, monitoring systems, and continued political support.

Page 36; Line 621: Scaling (i.e., sustainment & stabilization phase) was rarely achieved, suggesting that gamified PA interventions are still in its infancy, with a lack of proven effects (14), or a long timeline in scaling (22).

Page 37; Line 636: The overlap between adoption, implementation, and scaling observed in this study reinforces the value of combining EPIS and the Validated Scaling Instrument. Decisions made during exploration and adoption influenced implementation readiness, while implementation experiences shaped opportunities for sustainment and stabilization.

**R1-C3:**

- Can you clarify in your Theoretical Underpinning section why these two frameworks are necessary to include in the analysis? You have compared each of the steps of the frameworks and then what your resulting definitions were but why were these two frameworks selected to inform this process?

**Response:**

We thank the reviewer for this valuable comment. We agree that the rationale for selecting these frameworks was not sufficiently explicit in the original manuscript. We have revised the ‘Theoretical Underpinning’ section to clarify why both frameworks were included. Specifically, we explain that the EPIS framework was selected because it is a widely used implementation science framework that provides a structured understanding of implementation processes and the multilevel contextual factors influencing adoption, implementation, and sustainment. We further acknowledge recent work highlighting the need for greater attention to policy processes and institutionalization within implementation science frameworks, including EPIS (Crable et al., 2022). Therefore, because municipalities play a central role in translating local health initiatives into sustainable policy and practice, we complemented EPIS with the Validated Scaling Instrument. This framework was specifically developed to understand the scaling of public-sector innovations and provides explicit attention to emergence, institutionalization, and stabilization phases, as well as scaling in, scaling out, and scaling up. Together, these frameworks enabled us to examine both implementation determinants and scaling dynamics, which aligns with the study's aim of understanding how municipalities move from pilot implementation toward sustainable policy integration. We have added this explanation to the manuscript and included the new reference to Crable et al. (2022).

**Adaptation to manuscript (page 8; line 162):**

Two complementary frameworks are integrated to analyze how municipalities adopt, implement, and scale gamified PA interventions from early interest to routine policy and practice: The Exploration, Adoption/Preparation, Implementation, and Sustainment (EPIS) framework and the Validated Scaling Instrument.

The EPIS framework was selected because it is a widely used implementation science framework that captures implementation as a phased process and considers the multilevel contextual factors influencing adoption, implementation, and sustainment (33). However, recent scholarship has highlighted the need for greater attention to policy processes and institutionalization within implementation science frameworks, including EPIS (34). As municipalities play a central role in translating local health initiatives into sustainable policy and practice, implementation perspectives alone were insufficient for the purposes of this study.

Therefore, we complemented EPIS with the Validated Scaling Instrument, which was specifically developed to understand the scaling of public-sector innovations and distinguishes between emergence, institutionalization, and stabilization as scaling phases. The instrument also provides explicit attention to scaling strategies, including scaling in, scaling out, and scaling up, which are particularly relevant for municipalities seeking to move beyond pilot implementation toward broader institutional and policy integration (32). Together, these frameworks offer a comprehensive lens for understanding both implementation processes and scaling dynamics within municipal contexts.

**R1-C4:**

- General formatting and grammar check should be done - there were a few noted issues like on Line 275.

**Response:**

Thank you for this suggestion. We carefully reviewed the manuscript and corrected several minor formatting and grammatical issues throughout the text. In addition, we standardized the formatting of *'Missie Master'* throughout the manuscript.

**Reviewer #2**

**R2-C1:**

Very practicable and interesting paper regarding decision-making pathways for adopting, implementing, and scaling gamified physical activity interventions in municipal policy and practice in the Netherlands

**Response:**

We thank the reviewer for this positive assessment of our manuscript and for recognizing its practical relevance and contribution to understanding decision-making pathways for the adoption, implementation, and scaling of gamified physical activity interventions in municipal policy and practice. We appreciate the reviewer's encouraging feedback.

**Reviewer #3**

**R3-C1:**

Please briefly define what constitutes "generic company outreach" compared to "personalized requests" to help private partners understand exactly why their current emails are being ignored.

**Response:**

We thank the reviewer for this helpful suggestion. We agree that the distinction between generic company outreach and personalized requests could be made more explicit. In this study, “generic company outreach” refers to standardized communication (e.g., mass emails, generic promotional messages, or advertisements) that is not tailored to a municipality’s specific context, priorities, or policy goals. In contrast, “personalized requests” refer to communication that is adapted to the municipality’s identified needs, local challenges, and policy objectives, and explicitly demonstrates how the intervention may support these priorities. To improve clarity, we have added this explanation to the Results section and revised the relevant sentence accordingly.

**Adaptation to the manuscript (page 23; line 429):**

Requests from companies often arrive as generic outreach, such as standardized emails or promotional messages sent to multiple municipalities without reference to local priorities. These communications were perceived as less credible than personalized requests that explicitly addressed a municipality’s specific needs, policy goals, or local challenges, or recommendations from trusted colleagues within the municipality.

We also adapted Table 4: Personalized requests from the company tailored to municipal needs, local challenges, and policy priorities”; “Generic company outreach (e.g., standardized emails or promotional messages not tailored to the municipality)

**R3-C2:**

To set the stage, please move a brief functional description of the 'Missie Master' app (currently in Line 150) into the Introduction so readers understand the tool's core mechanics before reaching the Methods

**Response:**

We have added a functional description of the Missie Master app into the Introduction by stating that this app encourages children to complete neighborhood-based missions involving physical activities and rewards them through the app for their participation.

**Adaptation to the manuscript (page 7; line 151):**

This study uses a case study focusing on the digital gamified PA intervention ‘Missie Master’. ‘Missie Master’ was designed by 8D, a Dutch company specialized in design-oriented and co-creative projects. The app was developed to promote physical activity among children aged 8-12 years (S1) within their local neighborhood. It encourages children to complete neighborhood-based missions involving physical activities and rewards them through the app for their participation. The app enables primarily health professionals or implementers, such as NSCs, as well as other coaches or teachers to create these interactive PA missions (e.g., neighborhood scavenger hunts and quizzes).

**R3-C3:**

In the implementation results, please list 2–3 specific items such as a manual or a marketing toolkit—that participants felt were missing from the initial rollout.

**Response:**

We thank the reviewer for this helpful suggestion. We agree that providing concrete examples improves the practical relevance of the implementation findings. Although the need for implementation support materials was initially identified during the adoption phase, participants indicated that these materials indeed remained important throughout implementation, particularly for local marketing. We therefore revised the implementation section to provide specific examples, including communication and marketing toolkits with ready-to-use promotional materials (e.g., flyers, social media content, and example messages). We also added examples of good practices shared between municipalities. For instance, one municipality adopted the use of a mascot after observing this approach in another municipality, illustrating how practical examples can support local promotion and engagement during implementation.

**Adaptation to the manuscript (page 29; line 468):**

Several materials that were needed during adoption and preparation remained important throughout implementation, particularly for local marketing, such as a communication or marketing toolkit with ready-to-use promotional materials, such as flyers, social media content, and example messages. In addition, examples of good practices from other municipalities were considered useful. For instance, one municipality adopted the use of a mascot after seeing this approach successfully applied elsewhere, illustrating how practical examples could support local promotion and engagement during implementation.

We also adapted Table 6 accordingly: *Availability of communication and marketing materials*

**R3-C4:**

Please offer a brief interpretation of why health professionals felt resources were adequate while policymakers remained skeptical.

**Response:**

We thank the reviewer for this insightful suggestion. We agree that the difference in perceptions between policymakers and health professionals needs further interpretation. After reviewing our dataset, we have added a brief explanation to the manuscript suggesting that policymakers may have a broader overview of municipal budgets, competing policy priorities, and anticipated financial constraints, making them more cautious about the availability of resources for long-term implementation and scaling. In contrast, health professionals may evaluate resource adequacy primarily from an operational perspective, focusing on the resources available for day-to-day implementation activities.

**Adaptation to manuscript (page 31; line 513):**

However, clear divergences in perception emerged between health professionals and policymakers. For example, policymakers generally felt financial resources were insufficient, while health professionals considered them adequate. This difference may reflect the distinct roles of these stakeholders. Policymakers typically have greater insight into municipal budget constraints, competing policy priorities, and anticipated future financial pressures, which may make them more cautious about the long-term availability of resources. In contrast, health professionals may assess resource adequacy primarily from an operational perspective, focusing on the resources available for delivering and supporting the intervention in practice.

**R3-C6:**

In the "Sustaining adoption efforts" section, please clarify if the two municipalities that dropped out suggested how the app's age-appropriateness or marketing timelines could have been better handled.

**Response:**

We thank the reviewer for this suggestion. To provide a more concrete example, we added a sentence illustrating how gamification can simultaneously contribute to objectives within the sport, health, and youth domains, thereby supporting GALA's goal of integrated collaboration across policy sectors.

**Adaptation to manuscript (page 34; line 561):**
For example, gamification can simultaneously support sport goals (e.g., increasing physical activity participation), health goals (e.g., promoting healthy lifestyles and prevention), and youth goals (e.g., engaging children and adolescents through attractive activities). This shared objective can facilitate collaboration between NSCs, public health professionals, schools, and youth workers.

**R3-C7:**

Please suggest a specific strategy for better defining the "role distribution" between policymakers and health professionals to avoid the coordination hurdles identified in your study.

**Response:**

We thank the reviewer for this valuable suggestion. We agree that the discussion would benefit from a more concrete strategy. Our findings indicated that uncertainty about responsibility for adoption between policymakers and health professionals emerged already during the adoption phase and continued to affect implementation and scaling efforts. We therefore added a recommendation that municipalities explicitly define ownership and responsibilities early in the adoption process. For example, policymakers may take the lead in strategic decision-making, policy alignment, and funding, while health professionals may be responsible for operational implementation, participant engagement, and providing feedback from practice. Establishing these roles from the outset may help avoid coordination challenges and facilitate scaling.

**Adaptation to the manuscript (page 40; line 699):**

This challenge was already evident during adoption, where municipalities reported uncertainty about who should take responsibility for adopting the intervention. To address this, municipalities may benefit from explicitly defining ownership and responsibilities early in the adoption process. For example, policymakers may lead strategic decision-making, policy alignment, and funding, while health professionals may lead operational implementation, participant engagement, and feedback from practice. This may facilitate coordination and support subsequent implementation and scaling efforts, echoing earlier findings that role clarity is essential for collaborative health innovation (Schwarz et al., 2024).

**R3-C8:**

In the Limitations section please specify how the small number of participating municipalities might impact the generalizability of these findings across the broader Dutch municipal landscape.

**Response:**

We thank the reviewer for this suggestion. We agree that the implications of the small sample size for generalizability could be described more explicitly. We have revised the Limitations section to clarify that participating municipalities differed in their experiences and contexts, but may not fully represent the diversity of the broader Dutch municipal landscape. Consequently, barriers, facilitators, and decision-making pathways identified in this study may vary across municipalities with different sizes, resources, governance structures, and levels of experience with gamification.

**Adaptation to the manuscript (page 39; line 689):**

Third, the study involved a relatively small sample size, including only a few municipalities, a limited number of participants within municipalities, and only one company. Although the participating municipalities varied in size and experience with gamification, they may not fully represent the diversity of the broader Dutch municipal landscape. Municipalities differ in their resources, governance structures, policy priorities, and levels of experience with innovation, which may influence adoption, implementation, and scaling processes. Consequently, the barriers, facilitators, and decision-making pathways identified in this study may not be equally applicable across all Dutch municipalities. Furthermore, the findings should be interpreted within the specific Dutch policy context in which the study took place.
