# Supplementary material for: Decision-making pathways for adopting, implementing, and scaling gamified physical activity interventions in municipal policy and practice in the Netherlands
Source: PLOS Digit Health. 2026 Oct 26;5(10):e0001792. doi: 10.1371/journal.pdig.0001792 (PMC13704860; doi:10.1371/journal.pdig.0001792)
Supplement: Peer review history

# Peer review: Decision Letter 0

**DOI:** 10.1371/journal.pdig.0001792.r001

**Authors:** Shannon Freeman, Shuduo Zhou

29 May 2026

Response to Reviewers Revised Manuscript with Track Changes Manuscript **Journal Requirements:**

1. Please amend your detailed Financial Disclosure statement. This is published with the article. It must therefore be completed in full sentences and contain the exact wording you wish to be published.

i. Please clarify all sources of financial support for your study. List the grants, grant numbers, and organizations that funded your study, including funding received from your institution. Please note that suppliers of material support, including research materials, should be recognized in the Acknowledgements section rather than in the Financial Disclosure.

ii. State the initials, alongside each funding source, of each author to receive each grant. For example: "This work was supported by the National Institutes of Health (####### to AM; ###### to CJ) and the National Science Foundation (###### to AM)."

iii. State what role the funders took in the study. If the funders had no role in your study, please state: “The funders had no role in study design, data collection and analysis, decision to publish, or preparation of the manuscript.”

iv. If any authors received a salary from any of your funders, please state which authors and which funders.

2. Please send a completed 'Competing Interests' statement, including any COIs declared by your co-authors. If you have no competing interests to declare, please state "The authors have declared that no competing interests exist". Otherwise please declare all competing interests beginning with the statement "I have read the journal's policy and the authors of this manuscript have the following competing interests:"

3. We note that your Data Availability Statement is currently as follows: All materials underlying this article are available in Supporting Information.

Please confirm at this time whether or not your submission contains all raw data required to replicate the results of your study. Authors must share the “minimal data set” for their submission. PLOS defines the minimal data set to consist of the data required to replicate all study findings reported in the article, as well as related metadata and methods (https://journals.plos.org/plosone/s/data-availability#loc-minimal-data-set-definition).

For example, authors should submit the following data:

- The values behind the means, standard deviations and other measures reported;

- The values used to build graphs;

- The points extracted from images for analysis.

Authors do not need to submit their entire data set if only a portion of the data was used in the reported study.

If your submission does not contain these data, please either upload them as Supporting Information files or deposit them to a stable, public repository and provide us with the relevant URLs, DOIs, or accession numbers. For a list of recommended repositories, please see https://journals.plos.org/plosone/s/recommended-repositories.

If there are ethical or legal restrictions on sharing a de-identified data set, please explain them in detail (e.g., data contain potentially sensitive information, data are owned by a third-party organization, etc.) and who has imposed them (e.g., an ethics committee). Please also provide contact information for a data access committee, ethics committee, or other institutional body to which data requests may be sent. If data are owned by a third party, please indicate how others may request data access.

4. In the online submission form, you indicated that “No additional data are available in order to protect participant privacy. Additional information can be obtained from the corresponding author upon reasonable request, while ensuring participant privacy is protected.”.

All PLOS journals now require all data underlying the findings described in their manuscript to be freely available to other researchers, either

1. In a public repository,

2. Within the manuscript itself, or

3. Uploaded as supplementary information.

This policy applies to all data except where public deposition would breach compliance with the protocol approved by your research ethics board. If your data cannot be made publicly available for ethical or legal reasons (e.g., public availability would compromise patient privacy), please explain your reasons by return email and your exemption request will be escalated to the editor for approval. Your exemption request will be handled independently and will not hold up the peer review process, but will need to be resolved should your manuscript be accepted for publication. One of the Editorial team will then be in touch if there are any issues.

**Additional Editor Comments (if provided):** **Reviewers' Comments:**

**Comments to the Author**

1. Does this manuscript meet PLOS Digital Health’s publication criteria?

Reviewer #1: Yes

Reviewer #2: Yes

Reviewer #3: Yes

**********

2. Has the statistical analysis been performed appropriately and rigorously?

Reviewer #1: N/A

Reviewer #2: Yes

Reviewer #3: N/A

**********

3. Have the authors made all data underlying the findings in their manuscript fully available (please refer to the Data Availability Statement at the start of the manuscript PDF file)?

The PLOS Data policy

Reviewer #1: Yes

Reviewer #2: Yes

Reviewer #3: Yes

**********

4. Is the manuscript presented in an intelligible fashion and written in standard English?

Reviewer #1: Yes

Reviewer #2: Yes

Reviewer #3: Yes

**********

**Reviewer #1:**  The authors provide a detailed review of their experience implementing a gamified physical activity intervention in various Netherlands municipalities. The data provides insight into enablers/barriers of implementation for these applications and within these settings. I suggest that the article be accepted after the following minor revisions are addressed:

- The introduction introduces necessary concepts/frameworks, but consider organising this section with subheadings for the different concepts

- Can you clarify in your Theoretical Underpinning section why these two frameworks are necessary to include in the analysis? You have compared each of the steps of the frameworks and then what your resulting definitions were but why were these two frameworks selected to inform this process?

- General formatting and grammar check should be done - there were a few noted issues like on Line 275.

**Reviewer #2:**  Very practicable and interesting paper regarding decision-making pathways for adopting, implementing, and scaling gamified physical activity interventions in municipal policy and practice in the Netherlands

**Reviewer #3:**  Please briefly define what constitutes "generic company outreach" compared to "personalized requests" to help private partners understand exactly why their current emails are being ignored.

To set the stage, please move a brief functional description of the 'Missie Master' app (currently in Line 150) into the Introduction so readers understand the tool's core mechanics before reaching the Methods

In the implementation results , please list 2–3 specific items such as a manual or a marketing toolkit—that participants felt were missing from the initial rollout.

please offer a brief interpretation of why health professionals felt resources were adequate while policymakers remained skeptical.

In the "Scaling up" section , please provide a concrete example of how gamification aligns with a specific goal of the Healthy and Active Living Agreement, such as integrated collaboration.

In the "Sustaining adoption efforts" section , please clarify if the two municipalities that dropped out suggested how the app's age-appropriateness or marketing timelines could have been better handled.

please suggest a specific strategy for better defining the "role distribution" between policymakers and health professionals to avoid the coordination hurdles identified in your study.

In the Limitations section please specify how the small number of participating municipalities might impact the generalizability of these findings across the broader Dutch municipal landscape.

**********

what does this mean?). If published, this will include your full peer review and any attached files.

**Do you want your identity to be public for this peer review?**  If you choose “no”, your identity will remain anonymous but your review may still be made public.

For information about this choice, including consent withdrawal, please see our Privacy Policy

Reviewer #1: No

Reviewer #2: No

Reviewer #3: **Yes:**  Bala Nimmana

**********

**Figure resubmission:**

**Reproducibility:**

To enhance the reproducibility of your results, we recommend that authors of applicable studies deposit laboratory protocols in protocols.io, where a protocol can be assigned its own identifier (DOI) such that it can be cited independently in the future. Additionally, PLOS ONE offers an option to publish peer-reviewed clinical study protocols. Read more information on sharing protocols at https://plos.org/protocols?utm_medium=editorial-email&utm_source=authorletters&utm_campaign=protocols

# Author comment: Author response to Decision Letter 1

**DOI:** 10.1371/journal.pdig.0001792.r002

14 Jul 2026

Attachment Submitted filename: Response to Reviewers_.docx

# Peer review: Decision Letter 1

**DOI:** 10.1371/journal.pdig.0001792.r003

**Authors:** Shuduo Zhou

27 Sep 2026

Decision-making pathways for adopting, implementing, and scaling gamified physical activity interventions in municipal policy and practice in the Netherlands

PDIG-D-26-00195R1

Dear Prof. dr. van Bottenburg,

We are pleased to inform you that your manuscript 'Decision-making pathways for adopting, implementing, and scaling gamified physical activity interventions in municipal policy and practice in the Netherlands' has been provisionally accepted for publication in PLOS Digital Health.

Before your manuscript can be formally accepted you will need to complete some formatting changes, which you will receive in a follow-up email from a member of our team.

Please note that your manuscript will not be scheduled for publication until you have made the required changes, so a swift response is appreciated.

IMPORTANT: The editorial review process is now complete. PLOS will only permit corrections to spelling, formatting or significant scientific errors from this point onwards. Requests for major changes, or any which affect the scientific understanding of your work, will cause delays to the publication date of your manuscript.

If your institution or institutions have a press office, please notify them about your upcoming paper to help maximize its impact. If they'll be preparing press materials, please inform our press team as soon as possible -- no later than 48 hours after receiving the formal acceptance. Your manuscript will remain under strict press embargo until 2 pm Eastern Time on the date of publication. For more information, please contact digitalhealth@plos.org.

Thank you again for supporting Open Access publishing; we are looking forward to publishing your work in PLOS Digital Health.

Best regards,

Shuduo Zhou, Ph.D.

Section Editor

PLOS Digital Health

***********************************************************

**Additional Editor Comments (if provided):**

**Reviewer Comments (if any, and for reference):**

Reviewer's Responses to Questions

**Comments to the Author**

Reviewer #1: All comments have been addressed

**********

publication criteria?

Reviewer #1: (No Response)

**********

3. Has the statistical analysis been performed appropriately and rigorously?

Reviewer #1: (No Response)

**********

4. Have the authors made all data underlying the findings in their manuscript fully available (please refer to the Data Availability Statement at the start of the manuscript PDF file)?

The PLOS Data policy

Reviewer #1: (No Response)

**********

5. Is the manuscript presented in an intelligible fashion and written in standard English?

Reviewer #1: (No Response)

**********

Reviewer #1: (No Response)

**********

what does this mean?). If published, this will include your full peer review and any attached files.

**Do you want your identity to be public for this peer review?**  If you choose “no”, your identity will remain anonymous but your review may still be made public.

For information about this choice, including consent withdrawal, please see our Privacy Policy

Reviewer #1: None

**********
